# Solution-Processed Thin Film of a Novel Organic Charge-Transfer Complex for Near-Infrared Detection in Field-Effect Transistors

**DOI:** 10.1021/acsami.5c23996

**Published:** 2026-02-20

**Authors:** Maria Elisabetta Giglio, Tommaso Salzillo, Dean Kos, Carme Martinez-Domingo, Sergi Riera-Galindo, Jose Miguel Asensi, Simone D’Agostino, Elisabetta Venuti, Marta Mas-Torrent

**Affiliations:** † 54449 Institute of Materials Science of Barcelona (ICMAB-CSIC), Campus UAB, Bellaterra 08193, Spain; ‡ Department of Industrial Chemistry “Toso Montanari”, 9296 University of Bologna, Navile Campus Via Gobetti 85, Bologna 40129, Italy; § Department of Applied Physics, 16724 University of Barcelona (UB), Barcelona 08028, Spain; ∥ Department of Chemistry “Giacomo Ciamician”, University of Bologna, Bologna 40136, Italy

**Keywords:** charge-transfer complexes (CTCs), co-crystals, organic field-effect transistors (OFETs), solution shearing, near-infrared (NIR) photodetectors

## Abstract

Charge-transfer complexes (CTCs) have garnered considerable attention owing to their tunable electronic properties, which arise from the unique interactions between electron donor and acceptor molecules. However, reported fabrication methods remain largely restricted to single crystals produced via drop-casting and coevaporation or thin films prepared by cosublimation, thereby limiting their practical applicability. In this work, we successfully synthesized cocrystals of (Ph-BTBT-C_10_)­(F_4_TCNQ) with a charge transfer degree (ρ) of 0.19. More importantly, we demonstrated the deposition of these cocrystals as thin films in organic field-effect transistors (OFETs) using a low-cost, rapid, and scalable solution-shearing technique compatible with large-area fabrication. The resulting CTC thin films exhibited n-type semiconducting behavior and showed a pronounced response to infrared light at 1050 nm. The combination of a single-component active layer whose near-infrared (NIR) absorption band can be chemically tuned through donor–acceptor engineering with a scalable solution-based processing method highlights the promise of CTC-based OFETs for advanced IR detection and sensing applications. These results open new perspectives for the technological exploitation of CTCs, a class of materials long studied but rarely integrated into practical devices.

## Graphical abstract

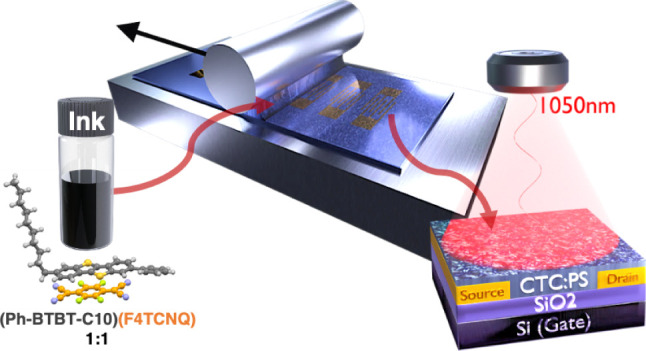

## 1 Introduction

Infrared (IR) detectors play a crucial role in next-generation optoelectronic devices, providing significant advantages in applications such as health monitoring,^1–4^ biomedical imaging,^5–7^ and night vision.^8^ Traditional IR detectors rely on inorganic phototransistors like silicon^9,10^ and indium gallium arsenide (InGaAs).^11,12^ They offer high performance but come with drawbacks, including rigidity, which makes them less suitable for flexible and lightweight applications as well as high cost and complex fabrication processes. In contrast, organic semiconductors (OSCs) are attracting attention due to their low cost, ease of processing, flexibility, and tunable optoelectronic properties.^13–16^

Most OSCs typically show a large band gap and hence can only respond to ultraviolet and visible light. However, organic charge-transfer complexes (CTCs) are particularly appealing candidates for IR photodetection due to their inherently narrow optical bandgaps.^4,8,17–24^ CTCs are composed of electron donor (D) and acceptor (A) molecules, where partial electron transfer from the highest occupied molecular orbital (HOMO) of the D to the lowest unoccupied molecular orbital (LUMO) of the A gives rise to new conduction and valence bands.^25–28^ This degree of charge transfer (ρ) is a critical factor that governs the electrical and optical properties of the CTCs. Additionally, the electrical characteristics of these materials are strongly influenced by their stoichiometry (D:A ratio) and the crystal packing. CTC cocrystals arrange by forming D/A segregated or mixed stacks, which significantly influence the material’s bandwidth and, consequently, their electronic characteristics. Thus, CTCs display diverse electrical behaviors, ranging from insulating^29^ and semiconducting^30–32^ to metallic^33^ and even superconducting,^29,34^ which are distinctive from those of their parent compounds.

One of the most compelling aspects of CTCs design is the opportunity for band engineering through careful selection of the D and A molecules. Previous work has demonstrated that various CTCs generate photocurrents for both holes and electrons, with the diffusion length of photocarriers being strongly influenced by the CT gap energy.^35^ However, despite their promising optical properties, the use of CTCs as active layers in organic field-effect transistors (OFETs) faces significant challenges. Current literature primarily reports CTCs produced as single crystals via solution methods (e.g., drop-casting) and coevaporation,^32,36–40^ or as thin films deposited through cosublimation techniques.^41–43^ These production methods limit scalability and hinder the practical implementation of CTCs for large-area applications, reducing their industrial relevance. The inherently low solubility characteristic of CTCs compared to their parent compounds poses serious difficulties for manufacturing thin films through solution-based processing techniques.^44–47^

Herein, we investigated a novel CTC formed by cocrystallizing the asymmetric donor molecule 2-decyl-7-phenyl[1]­benzothieno­[3,2-*b*]­[1]­benzothiophene (Ph-BTBT-C_10_) with the strong organic acceptor 2,3,5,6-tetrafluoro-7,7,8,8-tetracyanoquinodimethane (F_4_TCNQ). Ph-BTBT-C_10_ was selected due to its high hole mobility,^48–51^ while F_4_TCNQ was chosen for its strong electron affinity and ability to facilitate efficient charge transfer.^52–60^ Both families of materials have further been used for fabricating CTCs.^43,53,61^ Using the Bar-Assisted Meniscus Shearing (BAMS) coating technique, a high-throughput roll-to-roll compatible deposition method, we successfully fabricated large-area crystalline thin films of this CTC. The resulting films were integrated into OFETs, which exhibited robust n-type semiconducting behavior and a distinct response under 1050 nm irradiation, confirming their potential for Near-Infrared (NIR) photodetection. This work underscores the potential of CTCs as versatile materials for next-generation NIR sensing technologies by bridging fundamental material properties with scalable device fabrication.

## 2 Results and Discussion

Ph-BTBT-C_10_ and F_4_TCNQ were selected as D and A components, respectively, for the formation of CTC (Figure 1a). Ph-BTBT-C_10_ has been extensively studied as a high-mobility p-type organic semiconductor.^21,48,48–51,62–68^ Literature reports indicate that its HOMO energy level (−5.65 eV) is well-aligned with the LUMO level of F_4_TCNQ (−5.08 eV), supporting the feasibility of efficient CTC formation.^49,60^

**Figure 1:**
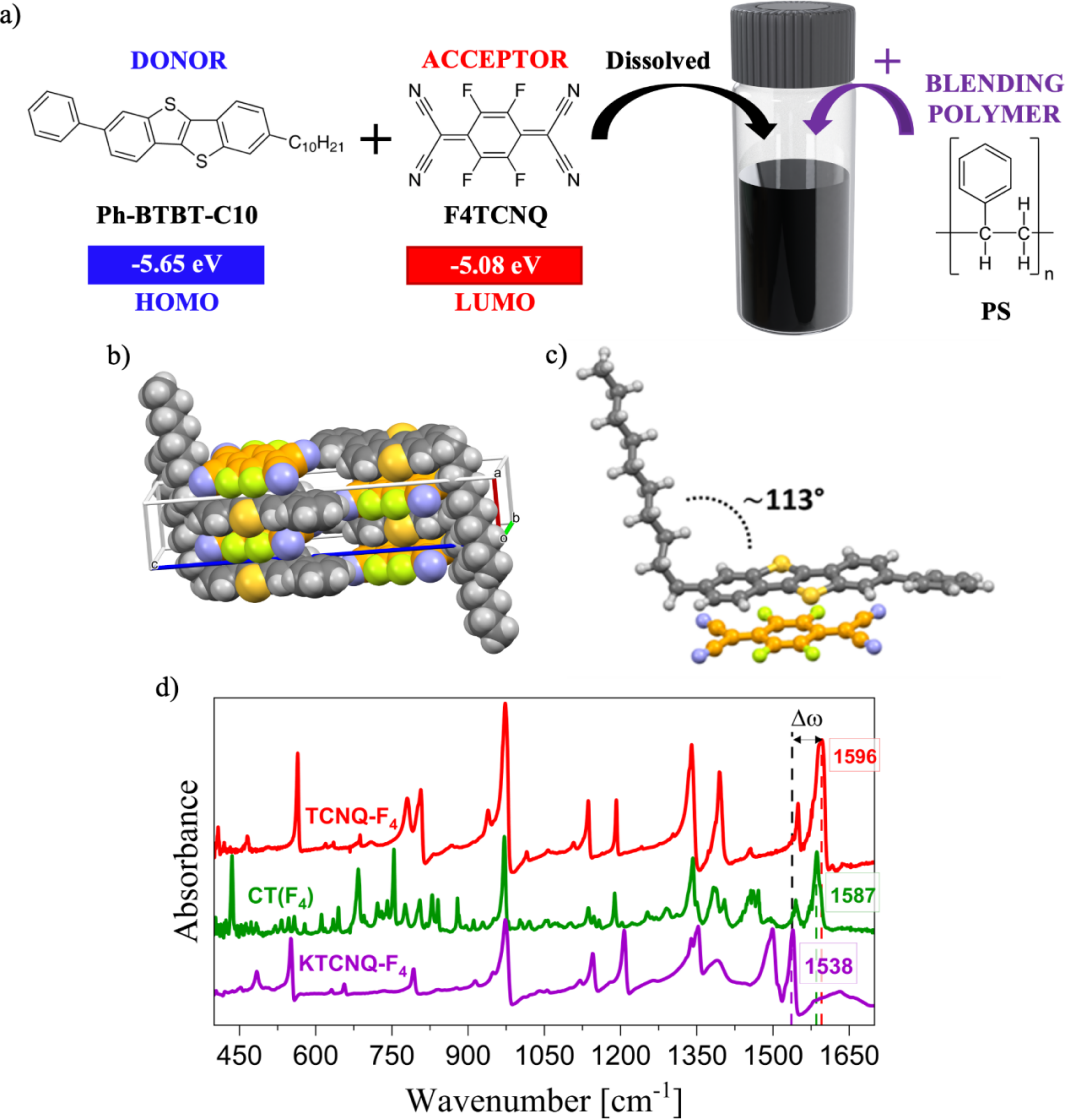
a) Molecular structures of the donor and acceptor materials, Ph-BTBT-C_10_ and F_4_TCNQ, respectively, along with their corresponding HOMO and LUMO energy levels reported in eV. b) Side view of the columnar stacking along the *a*-axis. Carbon atoms of Ph-BTBT-C_10_ and F_4_TCNQ are shown in gray and orange, respectively. Crystal packing structure of CTC (Ph-BTBT-C_10_)­(F_4_TCNQ). c) Detail of the molecular crystal structure of the CT complex. d) IR spectra of the CTC (Ph-BTBT-C_10_)­(F_4_TCNQ), the neutral F_4_TCNQ compound, and the ionic salt KF_4_TCNQ.

Single crystals of the (Ph-BTBT-C_10_)­(F_4_TCNQ) CTCs were successfully obtained in a 1:1 molar ratio using a mechanochemical approach, followed by slow solvent evaporation (see Experimental Section and Figure S1). Specifically, the donor and acceptor powders were ground together in a mortar to ensure intimate mixing, and the resulting mixture was dissolved in chlorobenzene:benzonitrile (5:1 v/v). Controlled solvent evaporation yielded the formation of very thin and interwoven needlelike single crystals exhibiting well-defined structural features.

The crystal structure of the charge-transfer complex (Ph-BTBT-C_10_)­(F_4_TCNQ), determined from powder X-ray diffraction (PXRD) data, is shown in Figure 1b,c (see also Figure S2). As reported in Table S1, the complex crystallizes in the triclinic *P*1̅ space group, with the asymmetric unit comprising one D molecule (Ph-BTBT-C_10_) and one A molecule (F_4_TCNQ). The parent compounds assemble into extended π-stacked columns along the *a*-axis. Within each stack, donor and acceptor molecules alternate in a regular ···A···D···A···D···D··· pattern (Figure 1b).

The interplanar distance between the Ph-BTBT-C_10_ and F_4_TCNQ molecules, measured from the molecular plane to the centroid, is approximately 3.3 Å. This value aligns with those reported for related CTCs featuring BTBT-based donor systems,^52,69,70^ while additional stabilization arises from weak hydrogen-bonding interactions between the CN groups of F_4_TCNQ and the aromatic C–H bonds of Ph-BTBT-C_10_ [NCN···C_CH_ = 2.685(1) Å].

As described in previous works,^71,72^ CTCs exhibit Raman and IR vibrations that are sensitive to the degree of charge transfer. The Raman spectra of (Ph-BTBT-C_10_)­(F_4_TCNQ) single crystals are shown in Figure S3 together with those of the parent F_4_TCNQ compound. Similar to previous works, clear shifts in the C=C double bonds of the central TCNQ core (around 1450 cm^–1^) and the stretching of the peripheral CN groups (around 2200 cm^–1^) are observed, in agreement with the formation of the CTC.^52^ However, in mixed stack, like the system studied in the present work, Raman spectra are dominated by the totally symmetric modes, whose frequency is affected by the interaction with the CT electrons (*e–mv* interaction).^73^ On the contrary, such a phenomenon does not affect the IR spectrum (Figure 1d), and hence, this represents a more appealing tool to estimate the CTC ionicity. As described by Girlando et al. and Bozio et al., C=C stretching modes move to lower energies on increasing the negative charge on the molecule following a linear relationship.^74,75^ Here, by comparing the spectra of the (Ph-BTBT-C_10_)­(F_4_TCNQ) CTC crystals with those of the neutral F_4_TCNQ compound and the fully ionized KF_4_TCNQ salt (Figure 1d), ρ was estimated to be 0.19 (see Appendix B in Supporting Information for calculation details).

Considering strictly the previously reported values for the Ph-BTBT-C_10_ HOMO and F_4_TCNQ LUMO energy levels, charge transfer should be unfavorable. However, it has to be taken into account that these values can be influenced by the technique used to extract them.^49,60,76^ In addition, in solid-state CTCs, the degree of charge transfer is not governed solely by HOMO–LUMO energy difference, since both donor and acceptor energy levels are affected by the environment through polarization and electrostatic interactions, which can stabilize the ionic state and enable partial charge transfer even when simple HOMO–LUMO alignment appears unfavorable.^25,58,77^ Additionally, ρ depends on the overlap of the broadened donor and acceptor density of states (especially tail states created by disorder and band dispersion), which determines how many states can participate in charge transfer at equilibrium, and on the donor–acceptor electronic coupling set by crystal packing.^25,38,78^

For the fabrication of the thin films, we prepared donor Ph-BTBT-C_10_ and acceptor F_4_TCNQ solutions in a 1:1 molar mixture (total concentration 18 mg mL^–1^) in chlorobenzene:benzonitrile (5:1 v/v). Three different approaches were tested for the preparation of the CTC inks: (1) dissolving the parent compounds separately and then mixing them (MET1), (2) premixing the powders in a vial before dissolution (MET2), and (3) using a mechanochemical approach consisting of manually mixing the powder of the parent compounds (MET3). Notably, solutions prepared using MET1 exhibited an orange color, while those prepared with MET2 and MET3 appeared black (Figure S4a), suggesting that CTC is already formed in the latter two methods. To gain insight into the electronic transitions and donor–acceptor interactions, UV–vis spectroscopy was performed on Ph-BTBT-C_10_, F_4_TCNQ, and (Ph-BTBT-C_10_)­(F_4_TCNQ) solutions (Figure S4b). Ph-BTBT-C_10_ exhibited a primary absorbance band centered at 327 nm, corresponding to π–π* electronic transitions within its aromatic cores, similar to the results reported in the literature.^21,62^ F_4_TCNQ displayed prominent absorption at 393 nm, also ascribed to π–π* transitions. This observation is consistent with previously reported data, confirming the characteristic electronic transitions of the TCNQ core.^79,80^ In the spectra of the (Ph-BTBT-C_10_)­(F_4_TCNQ) solutions, the absorption bands were red-shifted, especially the ones prepared with MET2 and MET3, indicating the presence of the D–A interactions. Moreover, the solutions prepared with MET2 and MET3 showed intense absorption bands in the NIR region (700–900 nm), corresponding to the charge transfer band.

These solutions were subsequently used as inks for thin-film deposition on SiO_*x*_ substrates with prepatterned electrodes employing the BAMS technique (Figure 2a; see Experimental Section for experimental details). To enhance processability and optimize film morphology, CTC-polymer blend inks were also prepared by mixing the CTC solutions with a polystyrene (PS) solution (18 mg mL^–1^) in the same solvent mixture at a fixed CTC:PS volume ratio of 4:1. PS was selected due to its low permittivity and proven role in improving film uniformity and overall device performance.^15,52,81,82^

**Figure 2:**
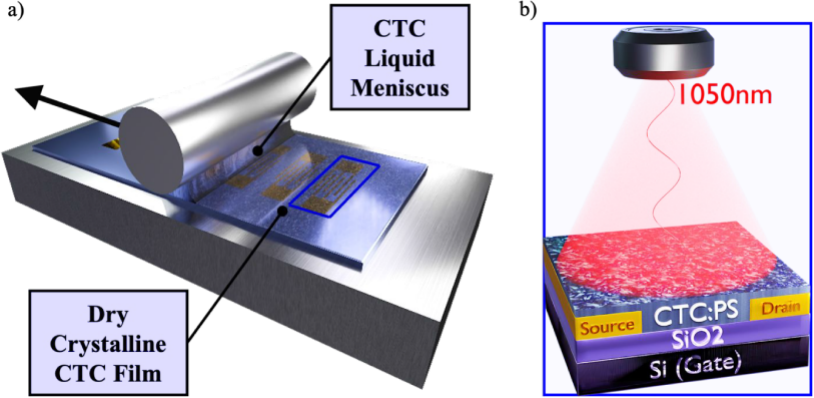
(a) Schematic of the Bar-Assisted Meniscus Shearing (BAMS) technique depicting the formation of a crystalline CTC film formed by dragging the meniscus of the CTC solution at a controlled speed. b) Scheme of the OFET device configuration, illuminated by an infrared light peaked at 1050 nm.

The films were characterized as active layers in OFETs, and their response to NIR light illumination was further investigated to elucidate the effects of photoexcitation on the device’s electrical characteristics (Figure 2b).

To achieve high-quality films, an extensive optimization process was carried out. Key parameters explored included coating speed and PS molecular weight, with the aim of producing uniform crystalline films with enhanced electronic properties. Details of the optimization procedure are provided in Appendix C of the Supporting Information. Film morphology and crystallinity were assessed using polarized optical microscopy (POM), atomic force microscopy (AFM), and X-ray diffraction (XRD). The optimal films, in terms of homogeneity, crystallinity, and mobility, were obtained using MET2 for solution preparation, a coating speed of 0.8 mm s^–1^ and a substrate temperature of 85 °C. Furthermore, the best device performance was achieved for films blended with PS of average molecular weight 10 kDa.

POM images of the optimized CTC-pristine and CTC:PS films are shown in Figure 3a,b. Both pristine and blended films present a similar polycrystalline morphology with small domains and no evident preferential in-plane orientation. However, the blended films display smaller domain sizes and a smoother surface compared to that of the pristine. This feature is further confirmed by the AFM topography images (Figure S5), which reveal small stripe-like crystal structures. The root-mean-square (RMS) roughness of the pristine CTC films is 25.45 nm, while the blended films exhibit a significantly lower roughness of 6.31 nm, indicating improved surface uniformity.

**Figure 3:**
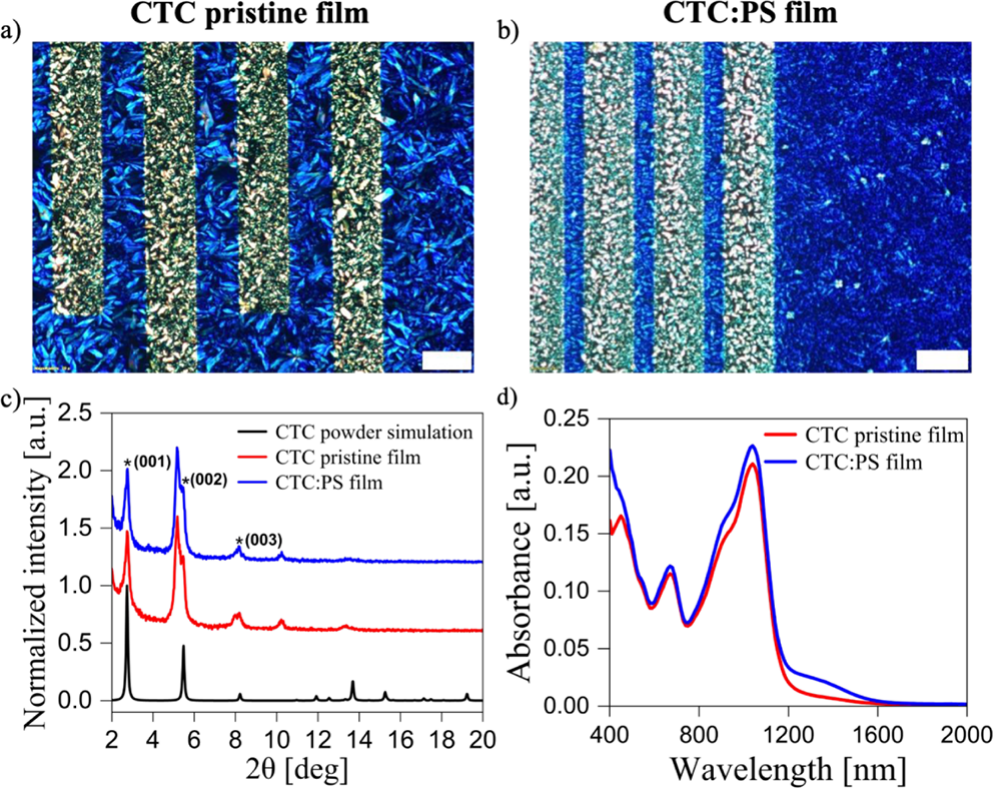
Cross-polarized optical microscopy images of (a) the pristine and (b) CTC blended films (white scale bar: 50 μm). (c) XRD characterization showing the normalized intensity for pristine CTC films and CTC blended with PS, together with the simulated pattern from the CTC-resolved crystal structure. (d) PDS absorbance spectra of pristine and blended CTC films.

For comparison, reference thin films of the individual components Ph-BTBT-C_10_ and F_4_TCNQ were also prepared. Ph-BTBT-C_10_ films exhibited large, well-defined birefringent domains under POM (Figure S6a, S6b), indicative of high crystallinity. This observation was further supported by AFM, which revealed extended crystalline domains (Figure S6c, S6d). In contrast, F_4_TCNQ films displayed small, disordered crystallites with regions of weak birefringence across the surface (Figure S6e, S6f), suggesting lower crystallinity and less uniform molecular alignment, as confirmed also by AFM (Figure S6g, S6h). XRD analysis was conducted to further examine the crystallinity and structural ordering of the films. The diffraction patterns of both pristine and PS-blended optimized CTC films are shown in Figure 3c. Both films exhibited similar diffraction patterns, consistent with the simulated pattern derived from the resolved single-crystal structure of the CTC. Distinct Bragg peaks, absent in spectra of the parent compound (Figure S7), were clearly observed and assigned to the 00*l* reflections of the cocrystal phase. From these measurements, the average interplanar spacing *d*_001_ was calculated to be 33.08 Å, closely matching the simulated value of 34.04 Å. Noticeably, in most measurements, two additional peaks were observed, which were attributed to the different orientations of the crystals.

To evaluate the optical absorption properties of the films, optimized thin CTC films were deposited onto glass substrates (Figure S8) and characterized using Photothermal Deflection Spectroscopy (PDS) (Figures 3d and S9).

PDS measurements revealed distinct absorbance features in the NIR region. In particular, a broad absorption band centered at 1038 nm was observed in both the pristine and PS-blended CTC films. This NIR band corresponds to the low-energy intermolecular charge-transfer transition and is a clear signature of CTC formation. Furthermore, the material’s HOMO–LUMO bandgap was estimated from a linear fit of the absorption edge, yielding 1.06 eV, in agreement with the values reported for similar CTCs.^52,83,84^ The FT-IR and Raman spectroscopy measurements of the thin films showed only the presence of CTC (Figure S10), confirming the chemical and physical homogeneity of the thin film prepared by BAMS.

The electrical performances of all fabricated devices were systematically evaluated (Table S2). Key performance metrics included charge carrier mobility, threshold voltage, hysteresis, and reproducibility. As previously mentioned, the best overall device performance was found in the film based on the CTC blended with PS 10 kDa and coated at 0.8 mm s^–1^ using MET2. Figure 4 reports the output and transfer characteristics of the optimized devices based on the blend and pristine CTC films.

**Figure 4:**
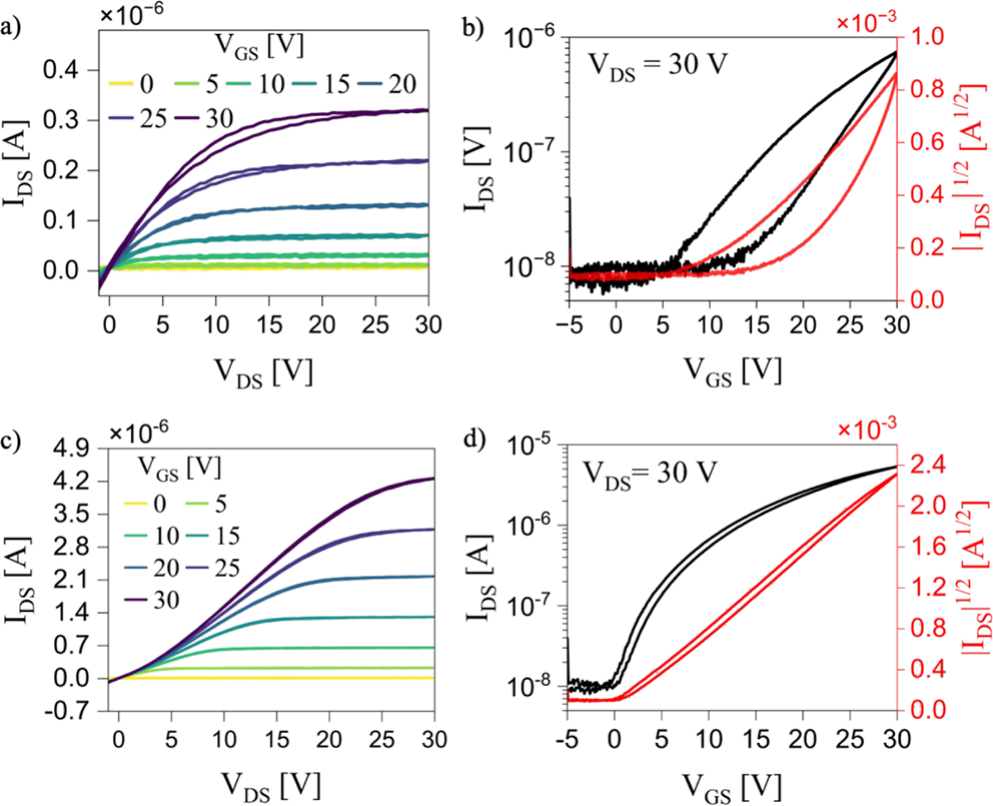
Electrical characterization under inert conditions of OFETs based on pristine CTC films (a,b) and CTC:PS (10 kDa) blended films (c and d). Panels (a) and (c) show output characteristics, while panels (b) and (d) display transfer characteristics in the saturation regime.

All devices exhibited n-type OFET behavior, consistent with previous reports on CTC based on BTBT derivatives.^54,55,61^ Notably, the pristine CTC films showed lower drain current, increased noise at higher gate voltages, and pronounced hysteresis in both output and transfer characteristics (Figure 4a,b), indicating inferior electrical performance. In contrast, the blended films demonstrated significantly higher drain current and improved transfer characteristics, including enhanced linearity in the square-root plots, threshold voltage closer to zero, and reduced hysteresis (Figure 4c,d). These improvements indicate enhanced charge carrier mobility and a lower density of interfacial charge trap states. This behavior may be attributed to the vertical phase separation occurring within the blend, which results in the formation of a PS layer at the dielectric/semiconductor interface. As previously reported, the PS layer effectively passivates interfacial charge traps, thereby improving charge transport.^85,86^

The optimized CTC-PS blended films achieved, in the saturation regime, the highest average electron field-effect mobility of (1.5 ± 0.3) × 10^–3^ cm^2^ V^–1^ s^–1^ and the lowest average threshold voltage of (2.3 ± 0.5) V. These devices also demonstrated superior reproducibility, as evidenced by the low standard deviation values in key performance metrics. In contrast, devices based on the pristine CTC films exhibited a lower average mobility of (5 ± 1) × 10^–4^ cm^2^ V^–1^ s^–1^ and a significantly higher threshold voltage of (12 ± 2) V. Moreover, control measurements of the blended Ph-BTBT-C_10_ films were performed in the same BGBC geometry, leading to an average hole electron mobility (in saturation) of 0.96 ± 0.08 cm^2^ V^–1^ s^–1^, consistent with the BTBT literature.^51,68^ The modest electron mobility extracted for the CTC films is consistent with values reported for many stoichiometric mixed-stack charge-transfer cocrystals,^43,55,87^ where charge transport is governed by the superexchange mechanism.^87,88^ In this class of materials, unipolar operation is also common, since orbital-symmetry/orthogonality penalties can suppress one carrier channel. Indeed, it has been reported that many TCNQ-derivative mixed-stack systems operate preferentially as n-type semiconductors.^87^

The shelf stability of the n-type CTC:PS blend-based OFETs was investigated under both inert and ambient conditions (Figure S11). Under an inert atmosphere, the devices maintained stable operation over a 4 week period, with negligible hysteresis and minimal threshold voltage shifts in the transfer curves, indicating excellent stability and reliability in controlled environments. However, devices exposed to air displayed hysteresis, a threshold voltage shift, and a reduction of the on/off ratio within 1 week, likely caused by interactions with oxygen and moisture that introduce trap states within the semiconductor layer.^89–94^

Building on these results, we next explored the NIR photoresponse of the optimized devices under inert conditions. A 1050 nm LED, closely matching the NIR maximum absorption band of CTC, was used as the excitation source. To identify the optimal geometry for maximizing photoresponse, we systematically varied the channel length (*L* = 25, 50, 100, 150, and 200 μm) and the length-to-width (*L*/*W*) ratio (including *L*/*W* = 0.01; *L* = 100 μm with *L*/*W* = 0.0056 and 0.0071; *L* = 25 μm with *L*/*W* = 0.0027, 0.0036, and 0.00625). The best photoresponse was obtained for a channel length of 25 μm and an *L*/*W* ratio of 0.0027. The transfer characteristics of the blended CTCs OFETs were measured in the saturation regime (*V*_DS_ = 30 V) under various light power densities (Figure 5a). The device exhibits a clear photoresponse in the on-state, which is reflected by an increase in *I*_DS_ accompanied by a threshold voltage shift toward negative values with increasing power density (Figure 5b).

**Figure 5:**
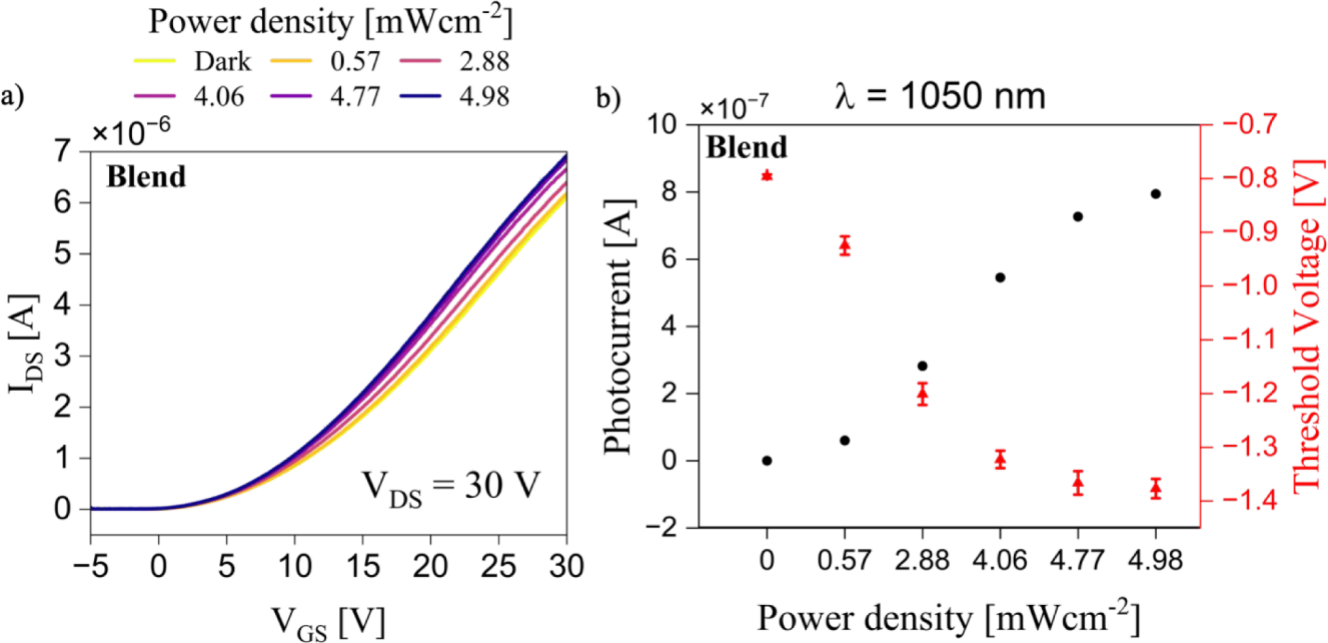
(a) Transfer curves for the blended CTC measured under varying light power densities using a 1050 nm LED. (b) Photocurrent (black, left axis) measured at *V*_GS_ = 30 V and threshold voltage (red, right axis) extracted from the same transfer characteristics in a) as a function of light power density.

This phenomenon is in agreement with the photovoltaic effect.^5,95–97^ Upon photon absorption, electron–hole pairs are generated, and while electrons (in an n-type OFET) migrate toward the drain electrode, contributing directly to conduction, holes accumulate and become trapped at the dielectric interface or at the source electrode. This results in a reduction of the potential barrier at the metal–semiconductor junction, and hence, more electrons are injected from the source into the channel, further enhancing carrier transport and leading to a drain current increase. Photoresponse measurements under continuous operation were also conducted across different operational states, including the off-state (linear and saturation regimes) and the on-state (linear and saturation regimes). In agreement with the transfer characteristics, only the on-state in saturation yielded a measurable response. Figure 6a illustrates the photocurrent response of both pristine and CTC:PS blended OFETs under pulsed illumination with the 1050 nm LED at varying light power densities ranging from 0.26 to 4.98 mW cm^–2^. A 5 s cycle is reported here, which was sufficient for the current to reach its maximum response. Additional measurements using 10 s cycles are reported in Figure S12 for both systems. Both pristine and blended devices exhibit a clear increase in drain current during illumination (ON) and return to baseline when the light is switched off (OFF).

**Figure 6:**
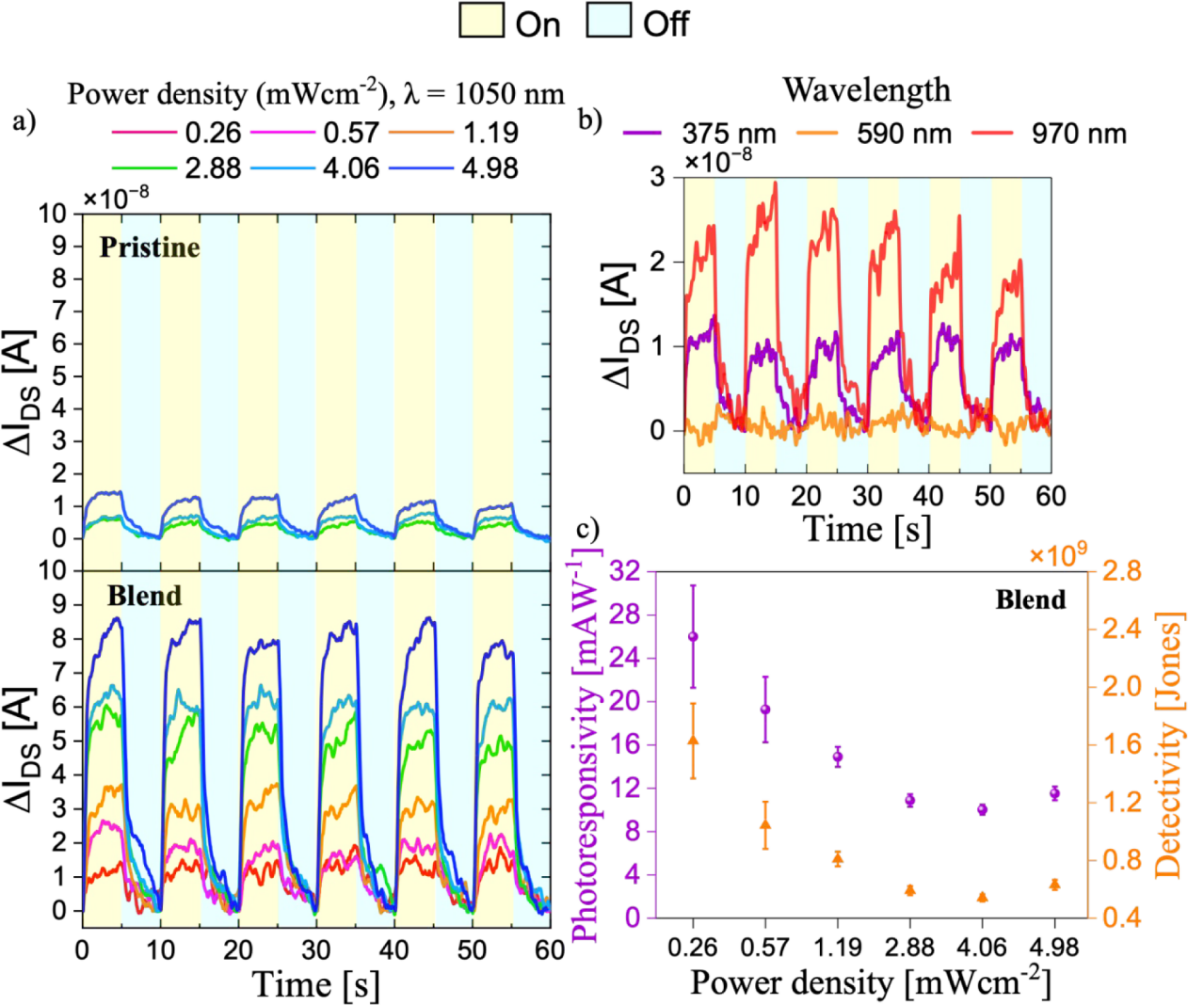
(a) Photocurrent response of pristine and blended CTCs upon the application of 5 s light pulses using LED light of 1050 nm. Yellow regions indicate illumination (ON), while light blue regions represent illumination off (OFF). Measurements were conducted at six light power densities: 0.26, 0.57, 1.19, 2.88, 4.06, and 4.98 mW cm^–2^. (b) Photocurrent response of the blended system under illumination applying 5 s illumination pulses using different LED wavelengths (375, 590, and 970 nm) with a fixed power density of 1.70 mW cm^–2^. (c) Responsivity (purple) and detectivity (orange) of the blended CTC OFET calculated as a function of light power density under 1050 nm illumination.

However, the pristine CTC-based devices display a weaker photocurrent amplitude and a limited dynamic response to increasing light intensity with no detectable response at light intensities below 1.19 mW cm^–2^. In contrast, the OFETs based on the blended films exhibit an enhanced photoresponse, with detectable photocurrents down to a light intensity of 0.26 mW cm^–2^. As illustrated in Figure 6b, we further evaluated the photoresponse of the CTC:PS blended film under LED illumination at wavelengths of 375 (UV), 590 (visible), and 970 nm (NIR), each at a fixed power density of 1.70 mW cm^–2^. However, the photocurrent generated at these wavelengths was significantly lower compared to the response observed at 1050 nm. At 970 nm, a reduced photocurrent may be attributed to a mismatch with the CTC’s maximum absorption peak. No detectable response was observed at 590 nm illumination, as CTC does not absorb in this spectral region. A modest photocurrent was recorded at 375 nm, consistent with previous studies showing UV-photoresponsivity in Ph-BTBT films.^21,62^ The lower photoresponse at this wavelength could be due to less efficient exciton dissociation or more trapping of the majority of charge carriers. The photoresponse selectivity at 1050 nm is a key feature for applications requiring wavelength-specific detection without the need for additional filters.

To quantify the photoresponse of the devices, the photoresponsivity (*R*) and specific detectivity (D*) of the OFETs were calculated across the studied range of optical power densities. The results of the CTC blended film are presented in Figure 6c, while data for the pristine CTC film are shown in Figure S13. Photoresponsivity reflects the device’s ability to convert incident light into photocurrent, while specific detectivity evaluates its sensitivity to weak light signals, taking into account noise interference. The blended films outperformed the pristine CTC devices in both *R* and D* by nearly 1 order of magnitude, highlighting the crucial role of the PS not only in enhancing OFET performance but also in improving the light detection efficiency.

As commonly observed in organic phototransistors, the responsivity decreased with increasing optical power density.^95,97–100^ The highest responsivity achieved by the blended devices was (26.4 ± 5) mA W^–1^, with a corresponding specific detectivity of (1.6 ± 0.3) × 10^9^ Jones, measured at the lowest tested light power density of 0.26 mW cm^–2^ (see Tables S3, S4, S5 for reference). Although comparison with literature data is not straightforward due to large differences in experimental setup, these photoresponse values are still below the state-of-the-art NIR-responsive phototransistors based on complex structures involving organic heterojunctions^2,98,101–113^ or conjugated donor–acceptor polymers or blends of polymers as active layers.^114–122^ However, reports on single-component NIR-phototransistors based on small molecules are very scarce,^109,111,112,123–125^ which can be attributed to the low intrinsic mobility of charge carriers in NIR-absorbing small molecules. Notably, the majority of the single-component systems reported exhibit absorption peaks in the region below 900 nm, i.e., closer to the visible–NIR edge rather than in the deep NIR region. In contrast, our system possesses a pronounced red-shifted absorption peak at nearly 1050 nm, highlighting its high potential as an NIR-responsive phototransistor. In this context, solution-processed thin films of small-molecule CTCs can offer new perspectives for the fabrication of transistors responding to selective NIR wavelengths by tuning the HOMO–LUMO bandgap of the CTCs by chemical design.

## 3 Conclusions

This work presents key advances in the design and application of CTC cocrystals for organic optoelectronics. After an extensive optimization process, homogeneous polycrystalline thin films of (Ph-BTBT-C_10_)­(F_4_TCNQ) and their PS blends were fabricated via scalable solution shearing, yielding OFETs with n-type transport. Blended films delivered enhanced performance considering mobility, trap density, and threshold voltage.

The intrinsically narrow bandgap of the CTC (1.06 eV) endowed the films with distinct NIR absorption. The resulting OFETs exhibited selective photoresponse at 1050 nm, demonstrating responsivity up to (26.4 ± 5) mA W^–1^ and specific detectivity of (1.6 ± 0.3) × 10^9^ Jones under 0.26 mW cm^–2^ illumination, in the case of the blended films. While device performance remains moderate compared to that of state-of-the-art NIR phototransistors, the use of a single, chemically tunable active layer provides a versatile platform for bandgap engineering via controlled charge-transfer modulation. Coupled with the compatibility of these materials with solution-printing, this approach enables scalable, low-cost fabrication of NIR photodetectors compatible with flexible substrates.

Overall, these findings validate the multifunctionality of CTCs and establish a benchmark for small-molecule, single-component, CTC-based NIR phototransistors, positioning them as promising candidates for next-generation IR detection and sensing technologies.

## 4 Experimental Section

### 4.1 Materials

2-Decyl-7-phenyl­[1]­benzothieno­[3,2-*b*]­[1]­benzothiophene (Ph-BTBT-C_10_) and 2,3,5,6-tetrafluoro-7,7,8,8-tetracyanoquinodimethane (F_4_TCNQ) were purchased from TCI, while polystyrene (PS) with molecular weights of 10, 100, and 280 kg/mol, along with 2,3,4,5,6-pentafluorothiophenol (PFBT), was obtained from Merck.

### 4.2 Single-Crystal Preparation

Single crystals of the CTC (Ph-BTBT-C_10_)­(F_4_TCNQ) were prepared by a liquid-assisted mechanochemistry method. The powders of the pristine components were weighed in the stoichiometric 1:1 molar ratio, transferred, and finely ground in a mortar, adding a few drops of a solvent mixture (5:1 toluene:acetonitrile), observing a sudden change in color which suggests the formation of the complex. To obtain single crystals suitable for an XRD investigation, the “as-synthesized” powder from the liquid-assisted mechanochemistry procedure was subjected to a recrystallization process in the same solvent mixture adopted for the synthesis. The powder of the complex was dissolved in toluene:acetonitrile 5:1, and after slow solvent evaporation, single crystals were successfully grown.

### 4.3 Solutions and Thin-Film Deposition

The donor Ph-BTBT-C_10_ and the acceptor F_4_TCNQ were mixed according to a 1:1 molar ratio and dissolved in chlorobenzene:benzonitrile 5:1 (concentration of 18 mg mL^–1^). Three methods were tested for preparing the CTC solutions. In the first method (MET1), the parent compounds were dissolved separately and then mixed. In the second method (MET2), the D and A powders were mixed in a vial and dissolved together. In the third method (MET3), cocrystals were synthesized by mechanochemistry, where the D and A powders were mixed using a mortar. PS (*M*_w_ = 10, 100, and 280 kg mol^–1^) solutions were also prepared (18 mg mL^–1^) in the same solvent mixture and mixed with the CTC solutions at a CTC:PS volume ratio of 4:1. The OSC inks were heated on a hot plate at 60 °C for 5 h, followed by sonication for 1 h. Subsequently, the solutions were heated to 85 °C and used as inks for film deposition via BAMS.

### 4.4 Device Fabrication

To fabricate Bottom-Gate-Bottom-Contact OFETs, we used heavily p-doped silicon wafers (Si/SiO*_x_*) with a total thickness of 525 ± 25 μm, which included a 200 nm SiO_2_ layer sourced from Si-Mat. The source and drain were designed as interdigitated electrodes with varying channel lengths (*L*) of 25, 50, 100, 150, and 200 μm, and *L*/*W* ratios of 0.00625, 0.0027, 0.0036, 0.0056, 0.0071, and 0.01. The electrode patterns were defined with a positive photoresist that was spin-coated onto the silicon wafers and exposed using a MicroWriter ML3 maskless photolithography system from Durham Magneto Optics Ltd.

After developing, the photoresist was developed, a 5 nm chromium (Cr) adhesion layer was deposited, followed by a 40 nm layer of gold (Au) using thermal evaporation, and then a liftoff process was performed. The resulting substrates were cleaned by sonication in acetone and isopropanol and subsequently dried with nitrogen. To improve charge injection, we chemically modified the work function of the gold electrodes with a self-assembled monolayer (SAM) of PFBT. Before the formation of the SAM, the substrates were exposed to UV-ozone for 25 min. The substrates were then immersed in a 15 mM PFBT solution in isopropanol for 15 min, followed by rinsing to remove any excess PFBT.

Subsequently, the heated CTC solutions were deposited by BAMS^45,52^ at 85 °C and at coating speeds of 0.8 mm s^–1^, 2 mm s^–1^, and 10 mm s^–1^. All fabrication steps were conducted under ambient conditions. We also deposited the thin films on glass substrates with a thickness of 0.13–0.16 mm to conduct spectroscopic FT-IR spectroscopy in transmittance mode as well as Photothermal Deflection Spectroscopy.

### 4.5 XRD Analysis and Film Characterization

Single-crystal data for the charge-transfer complex (Ph-BTBT-C_10_)­(F_4_TCNQ) were collected at 100 K on an Oxford XCalibur S CCD diffractometer equipped with a graphite monochromator (Mo Kα radiation, λ = 0.71073 Å) and a Cryostream 800 cryostat. Unfortunately, all single-crystal specimens tested consisted of weakly diffracting, interwoven thin needles that were difficult to analyze. Despite many attempts, only a triclinic unit cell could be determined, with the following parameters: *a* = 7.130 Å, *b* = 7.853 Å, *c* = 32.96 Å; α = 96.59°, β = 90.86°, γ = 106.19°; *V* = 1697.4 Å^3^. As a result, the structural solution of the compound was obtained by using powder X-ray diffraction (XRD) data. To this end, diffractograms were collected at RT on a Panalytical X’Pert PRO automated diffractometer equipped with a PIXcel detector and operated in transmission geometry (capillary spinner), using Cu Kα radiation without monochromator in the 2θ range 3°–70° (continuous scan mode, step size 0.0260°, counting time 889.70 s, Soller slit 0.02, antiscatter slit 1/4, divergence slit 1/4, 40 mA × 40 kV). Five diffraction patterns were recorded and summed to enhance the signal-to-noise ratio. Powder diffraction data were analyzed with the software EXPO2014,^126^ which is designed to analyze both monochromatic and nonmonochromatic data. Selected peaks were chosen in the 2θ range 10–50°, and a unit cell of ca. 1700 Å^3^ and with the most plausible space group *P*1̅ (*Z* = 2) was found using the algorithm N-TREOR09 ^127^ consistent with the previously indexed unit cell (see above) and with an asymmetric unit comprised of one F_4_TCNQ and one Ph-BTBT-C_10_ (CTC volume was evaluated to be approximately 890 Å^3^). For the preparation of the asymmetric unit, molecular fragments of F_4_TCNQ and Ph-BTBT-C_10_ were retrieved from the Cambridge Crystallographic Data Center;^128^ CSD refcodes are BAKPAE^129^ and ROQSAT,^64^ respectively. The structure was solved with a simulated annealing method without H atoms. To the so-obtained structural solution, H atoms were added in a calculated manner, and the structural model was optimized with MM and MOPac-PV7 ^130^ before the final Rietveld refinement. See Figure S2 for the pattern difference plot and Table S1 for the crystallographic details. The program Mercury^131^ was used for molecular graphics and to calculate intermolecular interactions in each crystal structure. Crystal data can be obtained free of charge via www.ccdc.cam.ac.uk/conts/retrieving.html (or e-mail: deposit@ccdc.cam.ac.uk); CCDC number 2487798.

Optical microscopy was conducted using an Olympus BX51 microscope equipped with polarizers and analyzers to capture detailed images of the thin films. Imaging was performed under both bright-field and cross-polarization conditions with two polarizer filters, at magnifications of 5×, 20×, and 50×.

XRD analysis was carried out using a Siemens diffractometer, utilizing Cu Kα radiation (wavelength: 0.1540560 nm) in a θ/2θ configuration to investigate the crystalline structure of the films.

Atomic force microscopy (AFM) imaging was performed with Park Systems NX10 in noncontact mode. The topographical data obtained were analyzed using Gwyddion software, which includes a specific tool for calculating the roughness and thickness of the films.

Infrared measurements (IR) were obtained with a PerkinElmer Spotlight 200i FTIR Microscope System with liquid nitrogen-cooled MCT detector coupled with a Spectrum 3 interferometer in transmission mode for single crystal and in reflection mode for thin film on a Si/SiO_*x*_ substrate. All measurements were a combination of 64 scans between 4000 and 600 cm^–1^ range and included background subtraction.

Raman spectra were obtained using a Horiba Xplora Plus spectrometer coupled with a BX-51 Olympus microscope to focus the laser on the sample with a 50× objective, an 1800 gr/mm grating, and a cooled charge-coupled device (CCD) as the detector. The laser power and exposure time were adjusted for each measurement to prevent sample damage. Typical integration times were 20 s at maximum power of 5–10 mW, with excitation at 532 nm from a continuous-wave solid-state laser.

Photothermal deflection spectroscopy (PDS) is a highly sensitive technique in which optical absorption is probed through the deflection of a laser beam. When the sample is heated by the excitation beam, the refractive index of the surrounding medium changes, producing a deflection proportional to the sample’s absorption. To enhance the sensitivity, the excitation light is mechanically modulated, enabling synchronous detection with a lock-in amplifier. In our case, PDS measurements were performed in the transverse configuration with the sample immersed in Fluorinert FC-40. A 10 mW He–Ne laser probe beam (632.8 nm) was directed tangentially across the sample surface, and its deflection was detected with a Hamamatsu C10442-02 position-sensitive detector connected to a Signal Recovery 7265 lock-in amplifier. The excitation light was provided by a 100 W tungsten–halogen lamp, dispersed by a PTI 01-0002 two-grating monochromator (covering the 400–2000 nm range), and mechanically modulated at 4 Hz with a Thorlabs MC1000 optical chopper.

### 4.6 Device Characterization

The OFETs were characterized by measuring their transfer and output characteristics using a Keithley semiconductor parameter analyzer connected to a Karl SÜSS probe station with all measurements performed under inert conditions. Output characteristics were measured with *V*_DS_ ranging from −5 to 30 V and *V*_GS_ from 0 to 30 V. Transfer characteristics were recorded in the saturation regime, *V*_DS_ = 30 V, with *V*_GS_ varying from −5 to 30 V. The electron field-effect mobility in saturation regime was calculated following the Shockley’s classic FET model.

1 $$\mu_{\mathrm{sat}}=\frac{2L}{C_{\mathrm{ox}}W}{\left(\frac{\partial \sqrt{I_{\mathrm{DS}}}}{\partial V_{\mathrm{GS}}}\right)}^{2}$$

where μ_sat_ represents the field-effect mobility in saturation, *W* and *L* are the width of the electrode and channel length, respectively, and *C*_ox_ is the capacitance of the dielectric per unit area. For SiO_2_ in our case, *C*_ox_ is 17.26 nF/cm^2^. *V*_GS_ refers to the applied gate-source voltage. A linear fit was employed to derive μ_sat_ and threshold voltage from the square root of the measured drain-source current (*I*_DS_) versus *V*_GS_, defined as

2 $$I_{\mathrm{DS}}=\frac{L}{W}\mu_{\mathrm{sat}}C_{\mathrm{ox}}{\left(V_{\mathrm{GS}}-V_{\mathrm{TH}}\right)}^{2},,V_{\mathrm{DS}}>\left(V_{\mathrm{GS}}-V_{\mathrm{TH}}\right)$$

Shelf stability was evaluated up to 4 weeks after the fabrication day, with transfer measurements conducted in saturation regime at *V*_DS_ = 30 V under inert conditions.

Light response performance was characterized using a custom-built setup equipped with four LEDs purchased from Thorlabs: LED370E (370 nm), LED590L (590 nm), LED970L (970 nm), and LED1050L2 (1050 nm). The LEDs were calibrated using a power meter and located at a distance of 2 cm from the OPTs. Transfer and *I*–*t* characteristics were measured at *V*_GS_ = 20 V and *V*_DS_ = 30 V under dark conditions and under irradiation at different power densities to assess the devices’ photoresponse. In order to measure the efficiency with which the device converts incident optical power (light) into an electrical signal, photoresponsivity (*R*) was calculated from the *I*–*t* measurements as

3 $$R=\frac{I_{\mathrm{light}}-I_{\mathrm{dark}}}{P_{\mathrm{opt}}\cdot A_{\mathrm{eff}}}$$

where *I*_light_ – *I*_dark_ represents the photocurrent, *P*_opt_ is the incident optical power density, and *A*_eff_ is the effective active area of the device. A higher photoresponsivity indicates a more efficient conversion of optical power into an electrical signal, which is important for the sensitivity of photodetectors in applications such as imaging, optical communication, and light sensing.

Assuming that the shot noise from dark current is the major contributor to the total background noise, the specific detectivity D*, a measure of the detector’s ability to detect weak signals, can be calculated as^5,132^

4 $$D^{*}=R\frac{A_{\mathrm{eff}}}{2e\cdot I_{\mathrm{dark}}}$$

where *e* is the elementary charge. The higher the specific detectivity, the more sensitive the device is to weak optical signals, with lower noise levels leading to higher specific detectivity.

Over 1,300 devices were fabricated and characterized throughout this study to guarantee statistical significance and reproducibility.

## Supplementary Material



## Notes

Additional experimental details, characterizations, and methods, including figures and tables (PDF)

## #

Institute of Microelectronics of Barcelona (IMB-CNM-CSIC), Campus UAB, 08193 Bellaterra, Spain
