# Supplementary material for: Solution-Processed Thin Film of a Novel Organic Charge-Transfer Complex for Near-Infrared Detection in Field-Effect Transistors
Source: ACS Appl Mater Interfaces. 2026 Feb 20;18(8):12889–901. doi: 10.1021/acsami.5c23996 (PMC12964347; doi:10.1021/acsami.5c23996)
Supplement: Supplementary file 1 [file am5c23996_si_001.pdf]

Supporting Information

Solution-Processed Thin Film of a Novel Organic Charge-Transfer Complex for Near-Infrared Detection in Field-Effect Transistors

Maria Elisabetta Giglio[1], Tommaso Salzillo[2], Dean Kos[1], Carme Martinez-Domingo[1#], Sergi Riera-Galindo[1], Jose Miguel Asensi[3], Simone D'Agostino[4], Elisabetta Venuti[2], Marta Mas-Torrent[1,]*

[1] Institute of Materials Science of Barcelona (ICMAB-CSIC), Campus UAB, Bellaterra 08193, Spain. E-mail: mmas@icmab.es

[2] Department of Industrial Chemistry "Toso Montanari", University of Bologna, Navile Campus, Via Gobetti 85, 40129, Italy.

[3] Department of Applied Physics, University of Barcelona (UB), Barcelona, 08028, Spain.

[4] Department of Chemistry "Giacomo Ciamician", University of Bologna, 40136, Italy.

[#] Current Affiliation: Institute of Microelectronics of Barcelona (IMB-CNM-CSIC), Campus UAB, 08193 Bellaterra, Spain.

Appendix A. Figures and tables

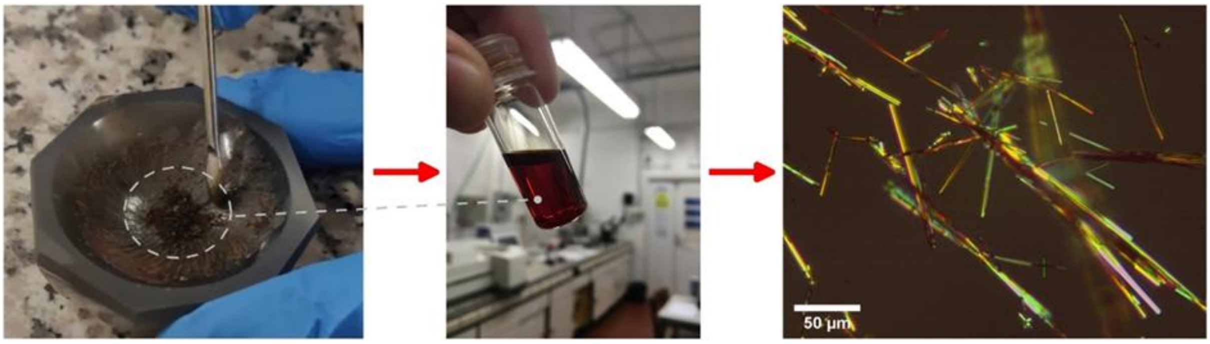

Figure S1. Illustration of the mechanochemical procedure used to create the (Ph-BTBT-C_{10})(F_4TCNQ) CTC single crystals. The process begins with the mixing in a mortar of the donor and the acceptor powders in a 1:1 molar ratio, followed by their dissolution in a 5:1 volume mixture of chlorobenzene and benzonitrile. Single crystals are then obtained through slow solvent evaporation.

Table S1. Crystal data and refinement details for crystalline (Ph-BTBT-C_{10})(F_4TCNQ).

	**(Ph-BTBT-C_{10})(F_4TCNQ)**
**Formula**	$C_{42}H_{32}F_4N_4S_2$
**FW**	732.84
**Temperature (K)**	300
**Cryst. System**	Triclinic
**Space group**	P-1
**Z**	2
**a (Å)**	7.0039(18)
**b (Å)**	7.988(2)
**c (Å)**	32.259(8)
**α (deg)**	96.860(10)
**β (deg)**	90.290(9)
**γ (deg)**	107.171(11)
**V (Å^3)**	1710.5(8)
**ρ_{calc} (g/cm^3)**	1.423
**μ (mm^{-1})**	0.192
**R_{wp} (%)**	4.8

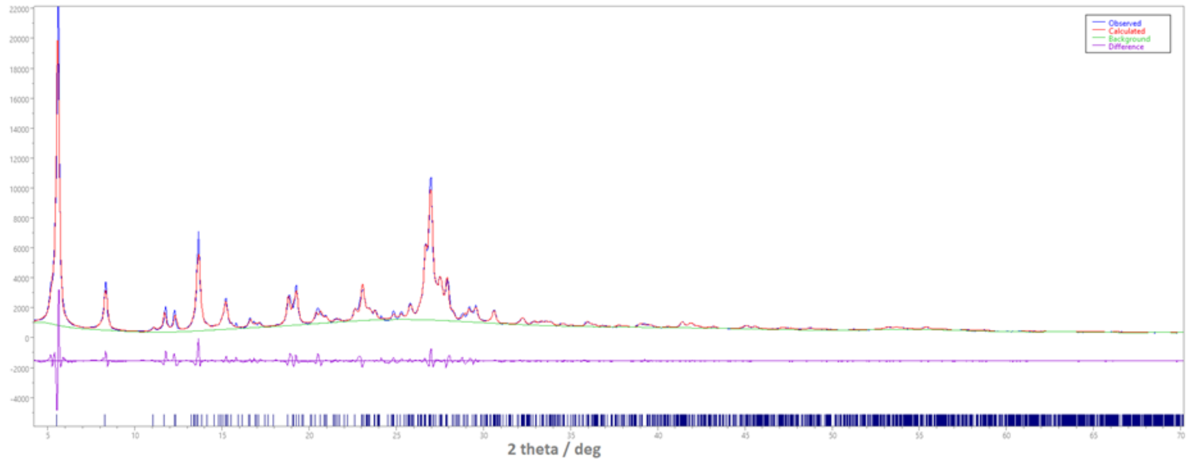

Figure S2. Powder XRD pattern of the CTC (Ph-BTBT-C_{10})(F_4TCNQ): experimental data (blue), calculated pattern from Rietveld refinement (red), and difference profile (magenta).

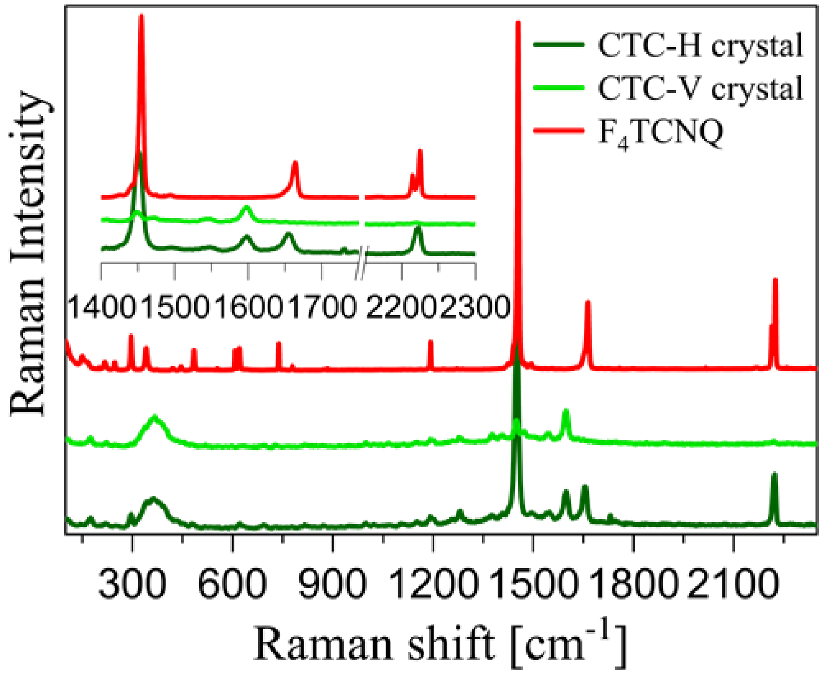

Figure S3. Raman spectra of the CTC-H crystal (dark green), compared with the spectra of CTC-V crystal (light green) and pure F_4TCNQ (red).

a)

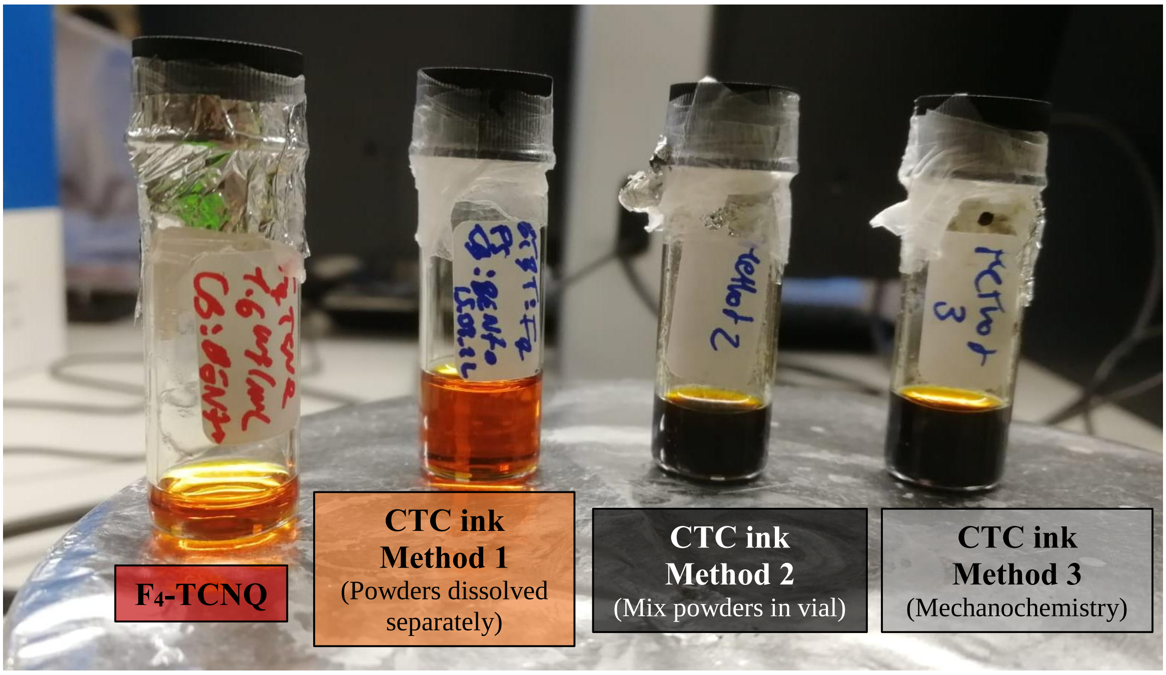

b)

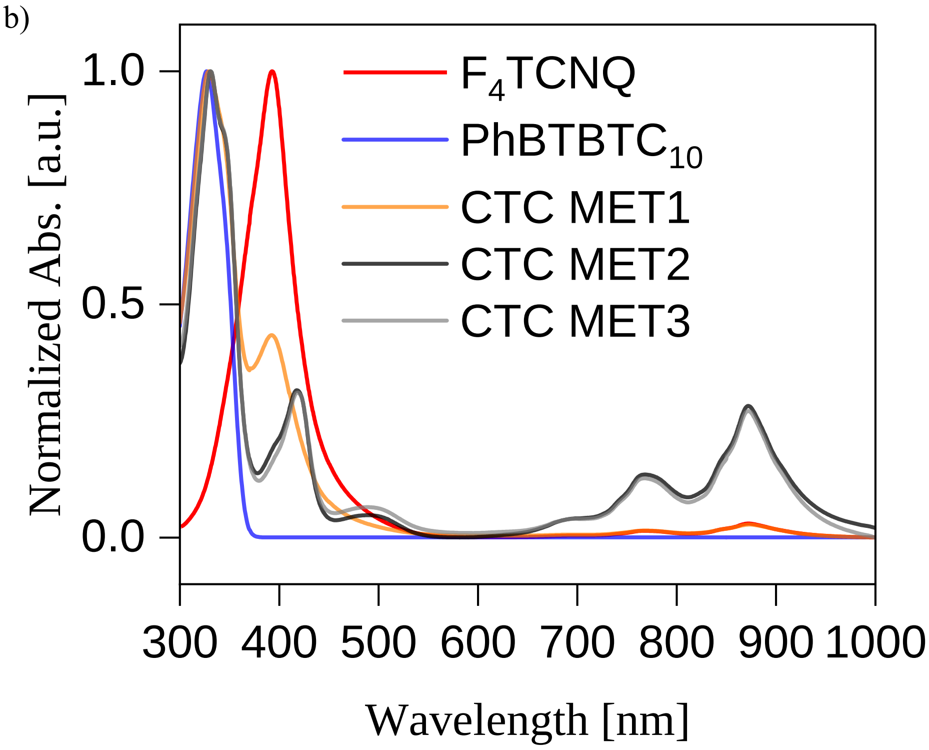

Figure S4. (a) Photograph of F_4TCNQ and charge transfer complex (CTC) (Ph-BTBT-C_{10})(F_4TCNQ) solutions (1:1 molar ratio) prepared using three methods: Method 1 (MET1), where F_4TCNQ and Ph-BTBT-C_{10} are dissolved separately and then mixed; Method 2 (MET2), where the powders are directly mixed in a vial and subsequently dissolved; and Method 3 (MET3), where the complexes are formed via mechanochemistry before dissolution. (b) UV-vis spectra of Ph-BTBT-C_{10} (blue), F_4TCNQ (red), and CTC solutions prepared via MET1 (orange), MET2 (black), and MET3 (grey).

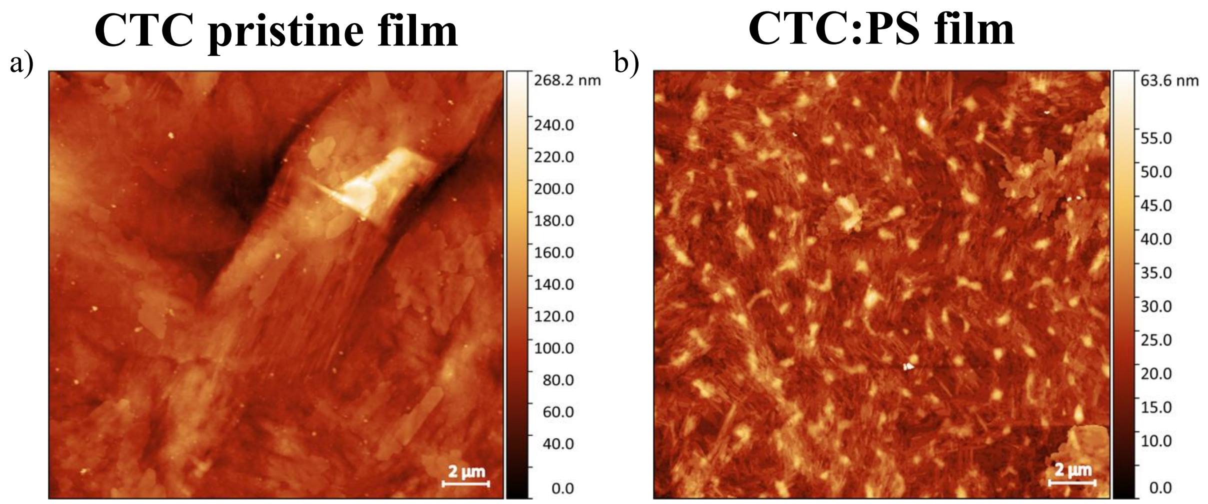

Figure S5. Atomic Force Microscopy (AFM) topography images of thin films deposited via BAMS at 0.8 mm/s. (a) Pristine charge-transfer complex (CTC) film, showing larger and irregular crystal structures. (b) CTC blended with 10 kDa polystyrene (PS) at optimized conditions, displaying smaller and more uniform crystal structures, indicative of improved film morphology.

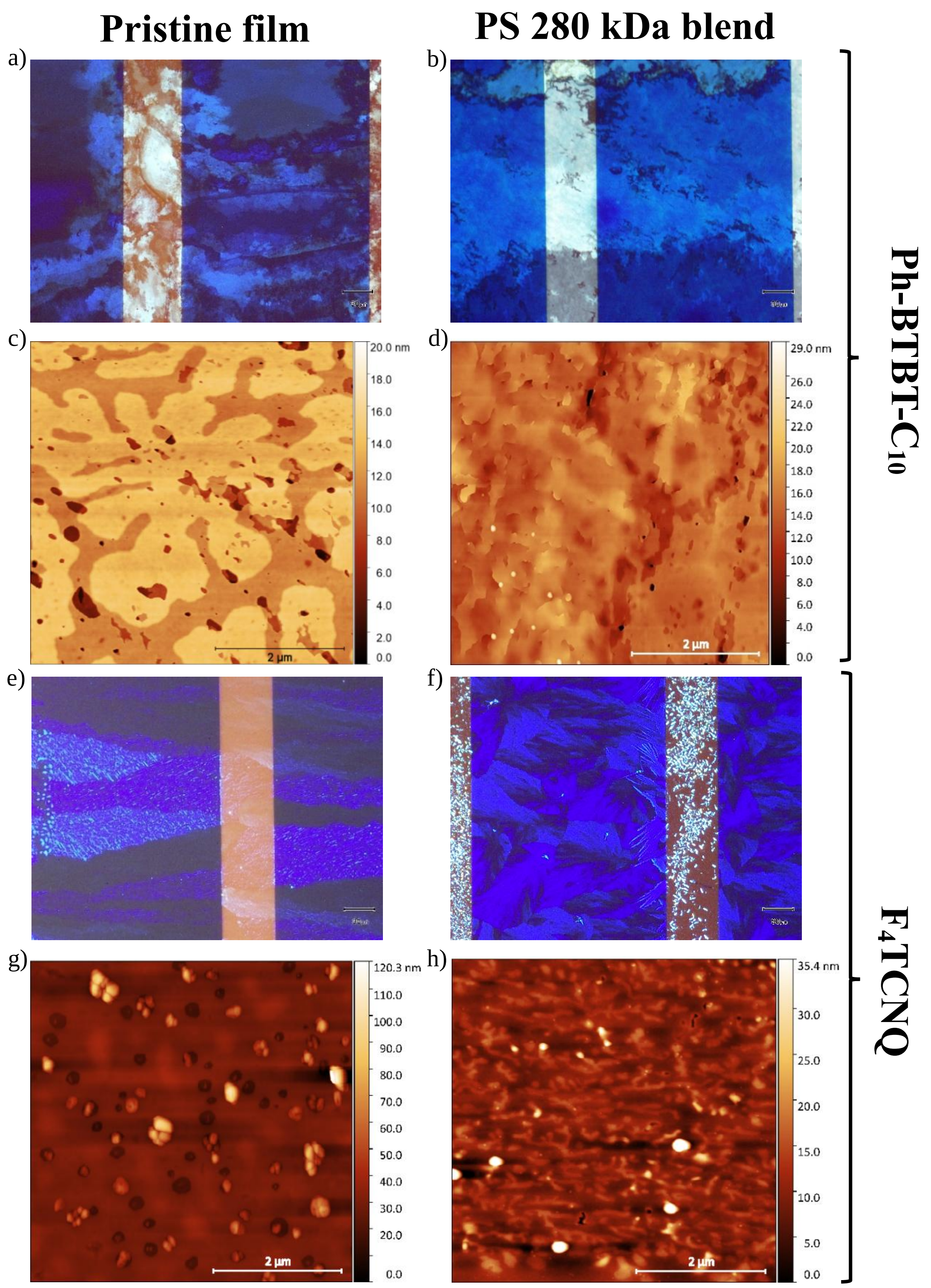

Figure S6. Comparative morphological analysis of films deposited via BAMS at 2 mm/s. Polarized Optical Microscopy (POM) images of Ph-BTBT-C_{10} thin films: (a) pristine and (b) blended with 280 kDa polystyrene (PS). (c, d) Corresponding Atomic Force Microscopy (AFM) topography images of the same films. (e, f) POM images of F_4TCNQ films: (e) pristine and (f) blended with 280 kDa PS. (g, h) Corresponding AFM images of F_4TCNQ films.

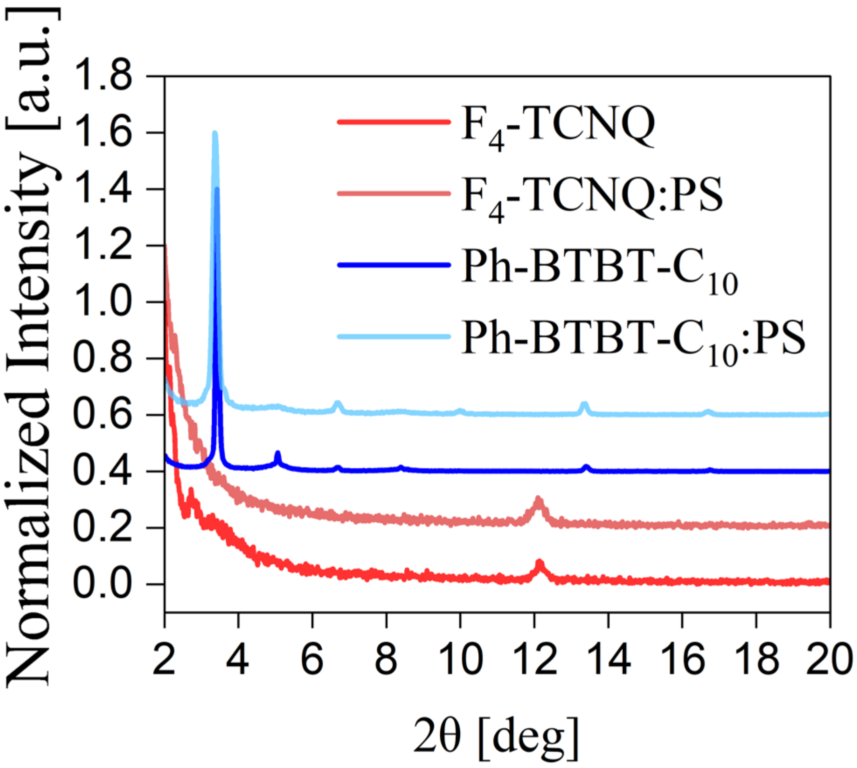

Figure S7. X-ray Diffraction spectra of the pristine and blended parent compounds Ph-BTBT-C_{10} and F_4TCNQ. The diffraction patterns reveal the crystalline nature of each compound, with distinct peaks corresponding to their respective lattice structures, serving as a reference for the characterization of charge transfer complex blended films.

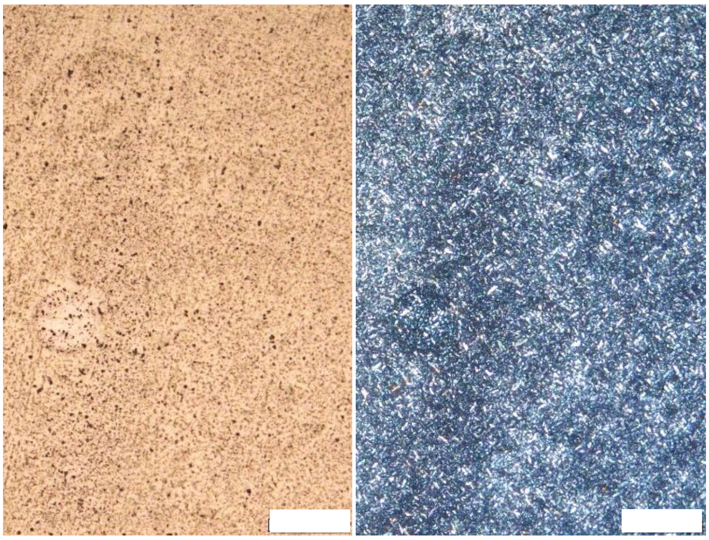

Figure S8. Optical microscopy images of a representative crystalline and homogeneous (Ph-BTBT-C_{10})(F_4TCNQ) film blended with polystyrene (10 kDa) deposited on a glass substrate at 0.8 mm/s for infrared transmittance measurements and photothermal deflection spectroscopy. (Left) Optical microscopy image in bright-field showing the uniformity of the film. (Right) Cross-polarized image under bright-field illumination, highlighting the crystalline nature and uniformity of the film.

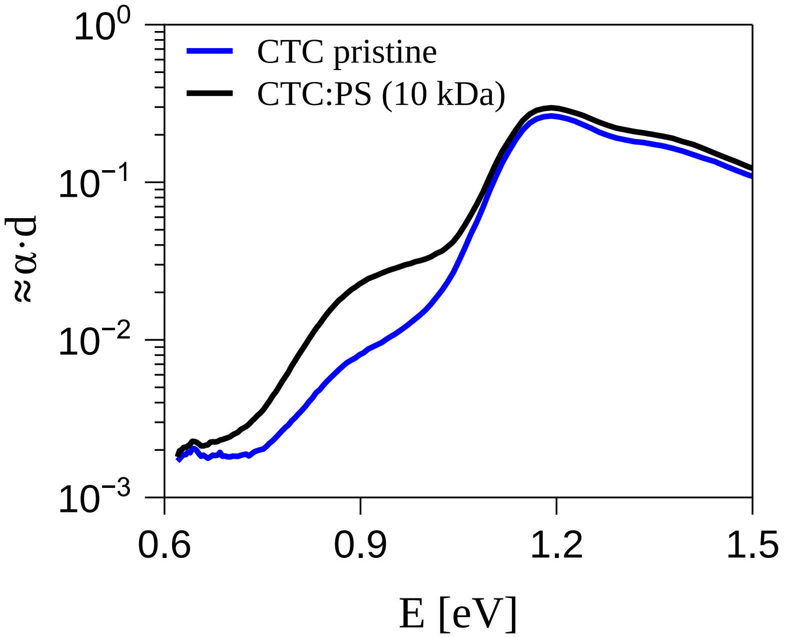

Figure S9. Photothermal Deflection Spectroscopy (PDS) of CTC films deposited on glass at 0.8 mm/s, showing the absorption edge of both pristine and blended CTC films. The product α·d, where α is the absorption coefficient and d is the film thickness, is plotted as a function of photon energy (eV). A visible charge transfer (CT) band around 0.9 eV is observed, indicating CT interactions in both pristine and blended films, with a clear electronic transition.

a)

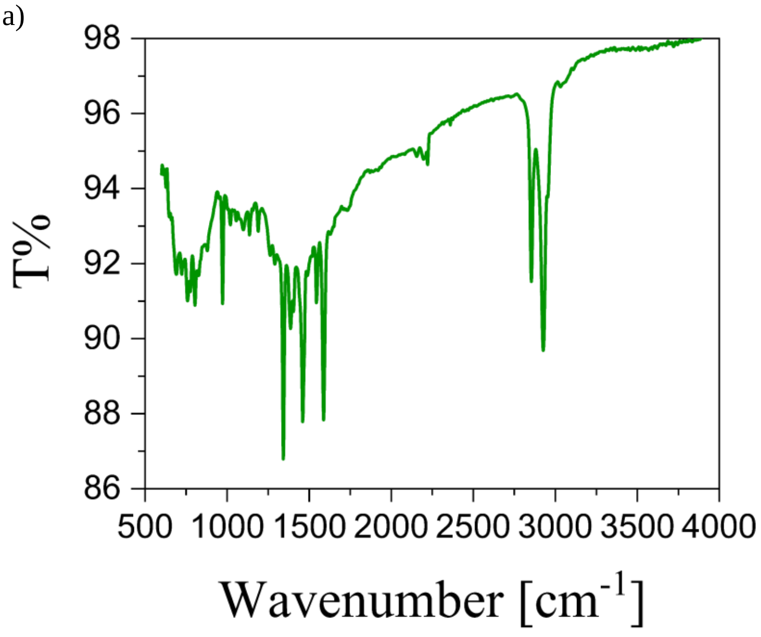

b)

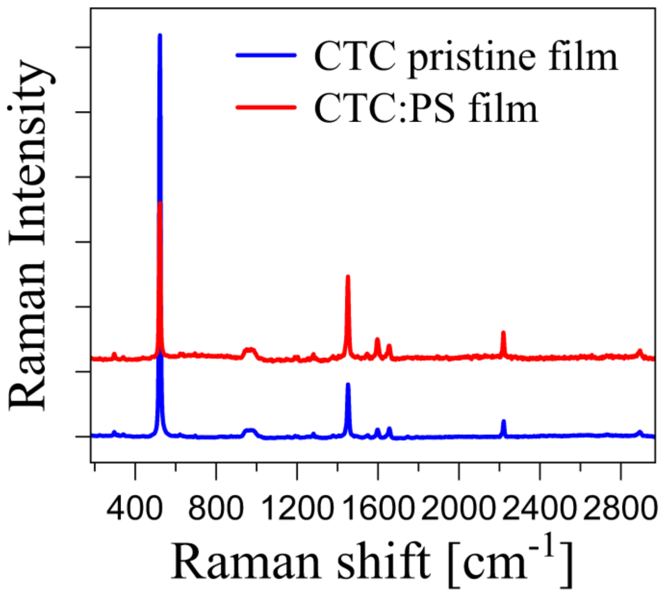

Figure S10. a) FTIR and b) Raman spectra of the CTC thin films, showing the presence of solely the charge-transfer complex, confirming the chemical and physical homogeneity of the thin film prepared by BAMS.

Table S2. Electrical parameters (mobility and threshold voltage) extracted from the electrical characteristics of the OFETs based on the charge-transfer complex (CTC) films deposited under different ink formulations and coating speed. Polystyrene (PS) of molecular weights 10 kDa, 100 kDa, and 280 kDa were used. The CTC inks were prepared using different methods (MET1, MET2, MET3). The most outperforming and reproducible OFET corresponds to CTC:PS (10 kDa), 0.8 mm/s, MET2, and is highlighted in green.

Formulation	Preparation method[1]	Deposition speed [mm s^{-1}]	Field-Effect Electron Mobility [cm^2 V^{-1} s^{-1}]	V_{th} [V]
CT:PS280K	1	2	Not working	
	2	0.8	$(3.6 \pm 0.6) \times 10^{-4}$	16 ± 3
		2	$(2.6 \pm 0.6) \times 10^{-4}$	12 ± 5
		10	$(3.5 \pm 0.4) \times 10^{-5}$	9 ± 6
	3	0.8	$(3.9 \pm 0.8) \times 10^{-4}$	18 ± 10
		2	$(9.6 \pm 0.7) \times 10^{-4}$	34 ± 4
		10	$(3.0 \pm 0.3) \times 10^{-4}$	22 ± 9
CT:PS100K	2	0.8	$(6.0 \pm 5.6) \times 10^{-4}$	0 ± 4
		2	Not working	
CT:PS10K	2	0.8	$(1.5 \pm 0.3) \times 10^{-3}$	2.3 ± 0.5
		2	$(2 \pm 1) \times 10^{-3}$	12 ± 4
CT pristine	1	2	Not working	
	2	0.8	$(5 \pm 1) \times 10^{-4}$	12 ± 2
		2	Not working	
		10	Not working	
	3	2	Not working	

[1] 1 indicates Method 1, 2 refers to Method 2 and 3 represents Method 3.

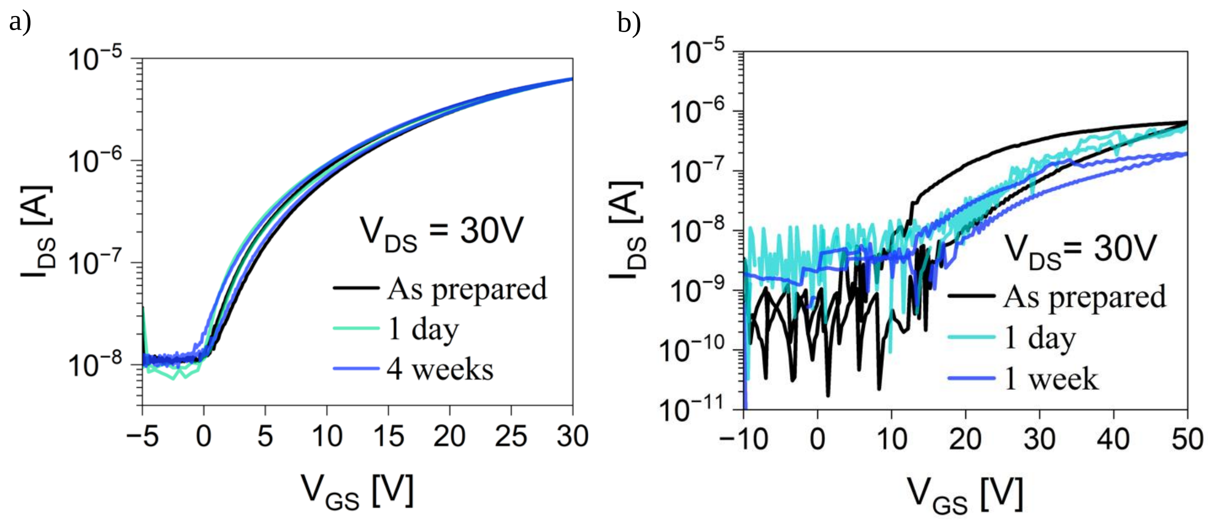

Figure S11. Shelf stability of CTC:PS (10 kDa) films (stored in a glovebox), with electrical measurements taken both in glovebox (a) and in air (b).

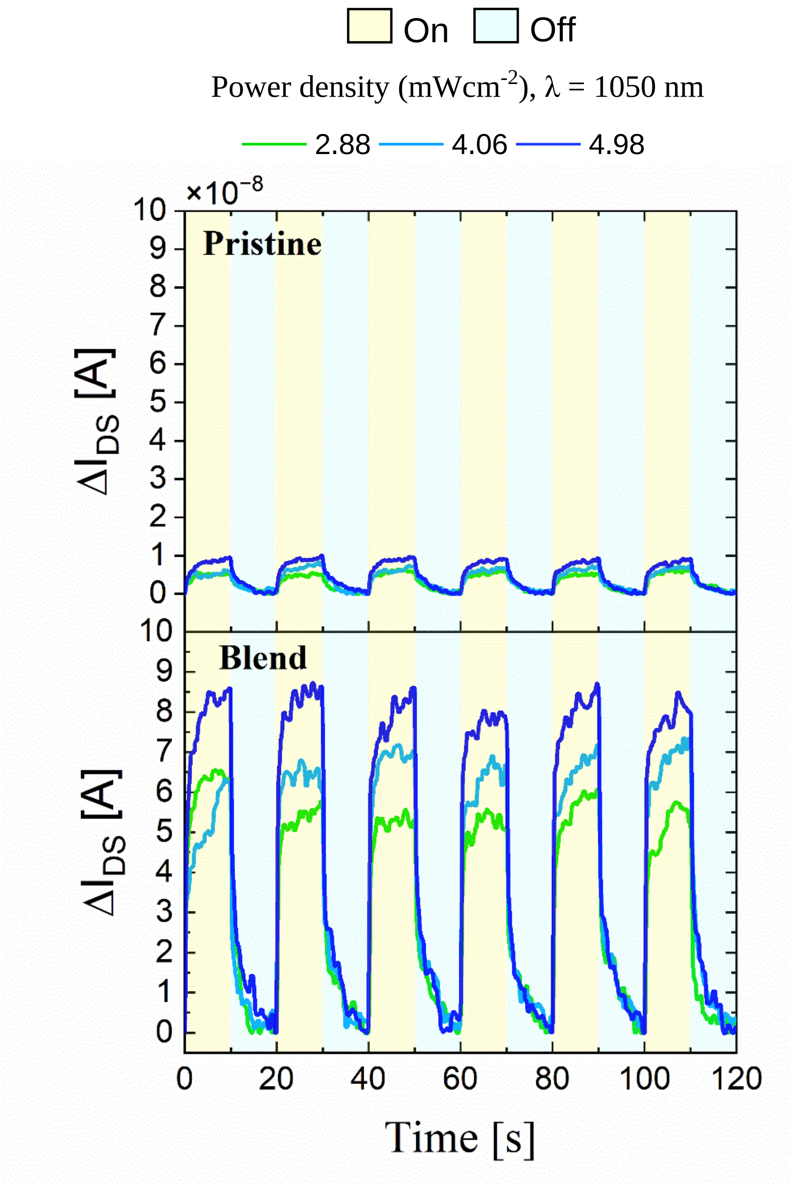

Figure S12. Photocurrent response of pristine and blended CTC films under illumination and dark cycles using LED light with a peak wavelength of 1050 nm. Yellow regions indicate illumination (ON), while light blue regions represent dark cycles (OFF). Measurements were conducted at three light power densities: 2.88, 4.06, and 4.98 mW cm^{-2}, with a 10-second interval between light ON and OFF states.

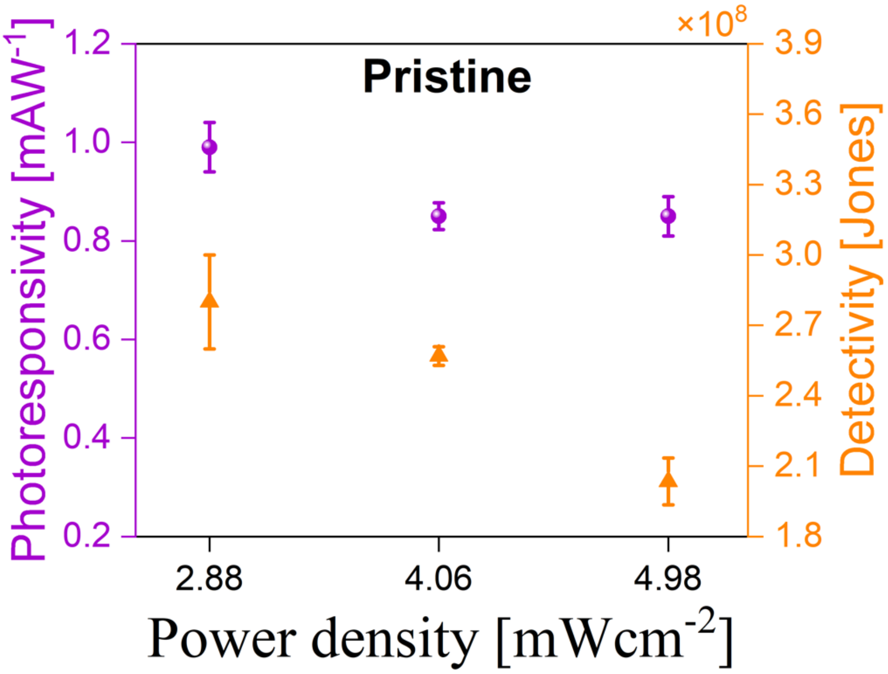

Figure S13. Responsivity (purple) and detectivity (orange) of the pristine CTC films-based OFETs, calculated as a function of light power density under 1050 nm illumination.

Table S3. Summary of reported near-infrared (NIR) organic phototransistors (OPTs) based on small molecules. All devices are fabricated on Si/SiO_2 substrates unless otherwise specified. Key parameters include class of active materials, deposition method, device architecture, average mobility, absorption range or peak, operational regime under illumination, light wavelength and power density, responsivity, and detectivity.

Year	Materials for active layers	Deposition	Device structure[1]	Field-Effect Mobility [$cm^2 V^{-1} s^{-1}$]	Absorbance [nm]	OFET State under illumination	Light Wavelength [nm]	Incident power density [$mWcm^{-2}$]	Responsivity [$A W^{-1}$]	Detectivity [Jones]	Ref
2023	CTC<br>MoO3: m-MTDATA (1:1)<br>+ HJ	Thermal Evaporation	BGTC	Ambipolar, linear<br>7.18×10^{-5} (n-channel)<br>6.60×10^{-5} (p-channel)	800-1600<br>Peak at 1100	ON, linear	1310	0.029	0.115 (n-channel)<br>2.6 (p-channel)	4.496×10^{9} (n-channel)<br>9.334×10^{10} (p-channel)	1
2022	CTC<br>DTTCNQ: 5,7-ICZ<br>(1:1)	Sublimation	BGTC	0.05 (ntype, saturation)	200-1300	ON, saturation	808	0.01 - 0.14	2.923 - 0.4	$(4.26 - 0.5) \times 10^{11}$	2
2022	CTC<br>TMTES-P:DFD<br>(1:2)	Drop-casting	BGTC	1.12 (ptype, saturation)	300-1000	OFF, saturation	700	0.74	776	5.49×10^{12}	3
							800	0.019	3.9×10^{4}	1.0×10^{14}	
							800	0.037	2.0×10^{4}	0.8×10^{14}	
							800	0.062	1.6×10^{4}	0.9×10^{14}	
							800	0.093	1.1×10^{4}	0.6×10^{14}	
							800	0.343	1.3×10^{3}	0.94×10^{12}	
							900	5.51	58	0.14×10^{11}	
							1000	4.18	135	0.93×10^{10}	
							1100	1.69	238	1.48×10^{10}	
							1200	0.32	650	5.50×10^{10}	
2021	CTC<br>PQT-12/F_4-TCNQ<br>+<br>semiconducting SWCNTs	Spin-coating	BGBC	Not given (p-type)	350-3200	ON, saturation	1000	$\sim 10^{-6}$	$\sim 10^{5}$	-	4
							1200	$\sim 10^{-6}$	$\sim 10^{4}$	-	
							1500	$\sim 10^{-6}$	$\sim 10^{6}$	-	
							2000	0.1	2.75×10^{6}	2×10^{12}	
2020	CTC<br>Graphene/TTF–CA (1:1)	Hot-dip coating	BGTC	Not given (p-type)	500-3000	ON, linear	2000	10^{-4}	30	-	5
2019	Graphene/P3HT:F4TCNQ (1:1)	Dip coating	BGTC	Not given (p-type)	600-1500	ON, linear	1000	4×10^{-9}	300	-	6
	Graphene/4T:F4TCNQ (1:1)						1000	1.4×10^{-9}	2000	-	
	Graphene/DH4T:F4TCNQ (1:1)						1100	1.2×10^{-9}	200	-	
2018	Graphene-TCNQ	Evaporation<br>+<br>Dip coating	BGBC	Not given (p-type)	From visible to 2000	ON, linear	1000	0.5×10^{-6}	2000	-	7
	Graphene-F_4 TCNQ							30×10^{-6}	120	-	
	Graphene-TCOQ							3×10^{-6}	240	-	
2018	TFT-CN	Solution epitaxy	BGTC	1.04 (ntype, saturation)	Peak at 830	ON, saturation	808	0.0025<br>0.1795	9×10^{4}<br>1.3×10^{3}	10^{14}<br>10^{12}	8
2018	SnPc	Thermal Evaporation	BGTC<br>BHJ	4.73×10^{-6} (ptype, saturation)	Peak at 890	ON, saturation	850	200	0.0106	2.88×10^{11}	9
	Pentacene/SnPc			3.85×10^{-3} (ptype, saturation)					1	3.12×10^{11}	
	Pentacene/SnPc/C60			1.77×10^{-3} (ptype, saturation)					2.7	1.41×10^{12}	
	Pentacene/SnPc/SnPc:C60			1.94×10^{-3} (ptype, saturation)					1.9	$1. \times 10^{12}$	
	Pentacene/SnPc:C60/C60			2.89×10^{-3} (ptype, saturation)					0.3	1.52×10^{11}	
	Pentacene/SnPc:C60			9.79×10^{-4} (ptype, saturation)					0.2	1.94×10^{11}	
2017	PTCDA:AlClPc:PbPc	Thermal Evaporation	BGTC<br>BHJ<br>C60/active layers	1.43×10^{-3} (ntype, saturation)	300-900	ON, saturation	850	29	0.58	1.5×10^{10}	10
2016	BODIPY-BF2	Spin-coating	BGTC	2.27×10^{-2} (ntype, saturation)	600-1000<br>Peak at 840	ON, saturation	760	0.5	196	-	11
							850		542	-	
							940		151	-	
2013	PbPc	Vacuum	BGTC<br>BHJ	$2.69 \times 10^{-5.}$ (ptype, saturation)	600-850<br>Peak at 750	ON, saturation	808	100	0.74×10^{-3}	-	12
	CuPc			7.17×10^{-4} (ptype, saturation)					44×10^{-3}	-	

[1] Bottom Gate Top Contact (BGTC), Bottom Gate Bottom Contact (BGBC), Bulk Heterojunction (BHJ)

Table S4. Reported near-infrared (NIR) phototransistors based on polymeric semiconductor systems. The table summarizes key performance metrics for Organic Field-Effect Transistors (OFETs) employing polymer blends or donor–acceptor systems as the active layer. Devices were fabricated primarily on Si/SiO_2 substrates unless otherwise noted. Various device architectures and light illumination conditions are presented, including hybrid structures, BHJs, and flexible formats. Performance parameters include field-effect mobility, absorption range, operating regime under illumination, light wavelength and intensity, responsivity, and detectivity.

Year	Materials for active layer	Deposition	Device structure[1]	Field-Effect Mobility [$cm^2\ V^{-1}\ s^{-1}$]	Absorbance [nm]	OFET State under illumination	Light Wavelength [nm]	Incident power density [$mWcm^{-2}$]	Responsivity [$A\ W^{-1}$]	Detectivity [Jones]	Ref
2022	P1:PC71BM	Spin-coating	BGBC	0.027 (ntype, saturation)	350-1000	OFF, saturation	850	0.063	968	1.8×10^{13}	13
	P2:PC71BM			0.01 (ptype, saturation)					118	1.3×10^{12}	
2021	Graphene/ZnO/PTB7-Th:IEICO-4F:PC71BM	Spin-coating	BHJ	ptype	760-1100	ON	488-1064	1.75×10^{-6} nW	~ 10^3	2.43×10^{13}	14
2021	PBTTQCN-TT:PC61BM	Spin-coating	BGTC BHS	pytpe	Broad up to 1880, Peak at 810	OFF, saturation	808	0.02	727	-	15
							1064	0.07	51	-	
							1550	0.07	123	-	
2021	DPPDTT:DCV3T Varying blending ratio	Spin-coating	BGTC	0.2 – 0.3 (ptype, saturation)	600 – 900	ON, saturation	740	Not clear	Few – 500	3×10^{11} - 2×10^{12}	16
							850	Not clear	250 - 2000	2×10^{11} - 10^{16}	
							940	Not clear	Few - 250	2×10^{11} - 10^{13}	
2021	P3HT/PolyTPD:BCF	Spin-coating	BHJ with ITO	7×10^{-3} (ptype, saturation)	up to $\lambda = \sim 3200$	ON, saturation	1500	0.072	0.538	-	17
							2000	0.058	0.541	-	
							3000	0.042	0.222	-	
2018	PBIBDF-BT	Spin-coating	Flexible OFET	3.49×10^{-2} (ntype, saturation)	808–980	ON, saturation	808	1	0.01	10^{10}	18
2016	PBDTT-DPP:PC71BM	Sputtering + Spin-coating	BGBC Hybrid BHJ	48–51 (ntype, saturation)	Peaks at 650 and 850	OFF, saturation	940	1	0 – 0.05	4×10^{10}	19
2015	P3HT:PEHTPPD-BT	Spin-coating	BG(ITO)TC OPTR BHJ	3×10^{-4} (ptype, saturation)	400-1100	ON, saturation	900	0.0014 – 0.0022	0.05	-	20
							1000		0.025	-	
2013	DPP-DTT:PCBM	Spin-coating	BGBC	0.3 (p-type, saturation)	400-1000	ON, saturation	808	67×10^{-6}	0.2	3.5×10^{12}	21

[1] Bottom Gate Bottom Contact (BGBC), Bulk Heterojunction (BHJ), Bottom Gate Top Contact (BGTC), Bilayer heterostructures (BHS), Indium Tin Oxide (ITO), Organic Phototransistor (OPTR)

Table S5. Reported organic photodiodes (OPDs) for near-infrared (NIR) detection based on polymeric active layers. The table compiles recent examples using donor–acceptor polymer blends or charge-transfer complexes, with device structures typically incorporating interfacial layers such as ZnO, MoO_3, PEDOT:PSS, or BPhen. Performance is summarized in terms of active materials, architecture, illumination wavelength and power, responsivity, and detectivity. Unless otherwise stated, devices are fabricated on Indium Tin Oxide (ITO)-coated glass substrates.

Year	Class of active materials	Materials for active layer	OPD Device structure	Light Wavelength [nm]	Power density [$mWcm^{-2}$]	Responsivity [$A\ W^{-1}$]	Detectivity [Jones]	Ref
2025	Polymers	PTB7-Th:ICS	ITO/PEDOT: PSS/active layer/ZnO NCs/Al	950	-	0.27	3.52×10^{11}	22
		PTB7-Th:Y6				0.01	1.29×10^{10}	
		ICS: PC61BM				0.27	1.09×10^{13}	
2024	Polymers	PCE10:DC4Cl	-	600 – 850	(10 - 11) × 10^3 mW	0.4	10^{13}	23
				1000		0.006	10^{11}	
				1050		0.0015	10^{11}	
		PCE10:BDP4Cl		600 - 1100		0.1	10^{13}	
				1300		0.003	10^{11}	
2024	Polymers	PTB7-Th/TTD(DTC-2FIC)2 (1:1, w/w)	ITO/ZnO/active layer/MoO3/Ag	1100	0.438	0.095	-	24
				1200		0.074	1.1×10^{11}	
2023	Polymers	PTB7-Th:L2	ITO/PEDOT:PSS/active layer/DPO/Al	1020	-	0.067	3.8×10^{11}	25
			ITO/ZnO/active layer /MoO3/Ag	1020		0.073	2.1×10^{12}	
			ITO/ZnO/active layer/MoO3/Ag	980		0.09	1.5×10^{11}	
			ITO/ZnO/active layer/MoO3/Ag	980		0.123	9.5×10^{9}	
			ITO/ZnO/active layer:Y6/MoO3/Ag	1020		0.084	2.9×10^{12}	
2023	Polymers	TQ-3T:IEICO-4F	ITO/active layer/MoOx/Ag	1200	100	0.05	1.03×10^{10}	26
		TQ-T:IEICO-4F				0.02	3.04×10^{8}	
2022	Polymers	P1:Y7	ITO/PEDOT:PSS/active layer/C60/Ag	1200	1	0.26	2.96×10^{10}	27
2022	Polymers	PTB7Th:COTIC-4F:Y6	4 different architectures	870	-	0.40 - 0.42	10^{10} - 10^{13}	28
				960				
				1060				
2021	Polymers	PCE10:COTIC 4Cl:PC71BM	ITO/PFNBr/active layer/LiF/Al	1100		0.3	5×10^{12}	29
2020	Polymers	PIF:ITIC-4F	ITO/ZnO/active layer/MoOx/Ag	600 – 750	< 12 mW	0.170 - 0.229	-	30
		PIF:IDFBR		600		0.009	-	
2019	Polymers	PTB7-Th:CO1-4Cl	ITO/ZnO/active layer/MoOx/Ag	>1000		<0.3	10^{10} – 10^{12}	31
2013	Polymers	PTZBTTT-BDT:PC61BM	ITO/PEDOT:PSS/active layer /Al	800	3.28	0.08	8×10^{12}	32
2013	Metal oxides	CTC SubPc/pristine MoO3	ITO/active layer/4, 7-diphenyl-1,10-phenanthroline BPhen/Al	870	50	0.000002	-	33
				>1000		10^{-7} – 10^{-6}		
		CTC BMO/fullerene		870		0.012		
				>1000		0.0007		

Appendix B.
Details on calculation of the degree of charge transfer

The degree of charge transfer $\boldsymbol{\rho}$ can be calculated by a simple proportion for each diagnostic vibration v:

$$\rho = \frac{\upsilon_{CTF_4} - \upsilon_{TCNQF_4}}{\upsilon_{KTCNQF_4} - \upsilon_{TCNQF_4}} \qquad \text{(S1)}$$

From IR spectra, we identified two out of the three diagnostic bands (usually used to estimate the ρ values) measured in neutral TCNQ-F_4 at 1595 and 1394 cm^{-1}. The same bands in the potassium salt, which is assumed to have $\boldsymbol{\rho}$ = 1, are shifted at 1538 and 1353 cm^{-1}. In the charge-transfer complex CT-F_4 the same modes are found at 1587 and 1384 cm^{-1}. By averaging the value calculated for each band (**Table S6**), a mean charge-transfer degree $\boldsymbol{\rho}$ of 0.19 is obtained.

Table S6. Diagnostic frequencies of TCNQ-F_4, CT-F_4 and KTCNQ-F_4 for the evaluation of the charge-transfer degree (ρ).

	v_1 (cm^{-1})	v_3 (cm^{-1})
**TCNQ-F_4**	1595	1394
**CT-F_4**	1587	1384
**KTCNQ-F_4**	1538	1353
$\boldsymbol{\rho}$	0.14	0.24

Appendix C.
Optimization of thin-film deposition

To achieve highly homogeneous crystalline films with enhanced performance, we conducted an extensive optimization of deposition parameters and ink formulations. Thin films of pristine and blended (Ph-BTBT-C_{10})(F_4TCNQ) were prepared using an equimolar ratio of Ph-BTBT-C_{10} and F_4TCNQ dissolved in a chlorobenzene:benzonitrile (5:1) solvent mixture. The optimization process included variations in the method used to produce charge-transfer complexes (CTCs), deposition speeds (0.8, 2, and 10 mm s^{-1}), and the average molecular weight of polystyrene (PS) (280 kDa, 100 kDa, and 10 kDa). To assess the morphology and crystallinity of the CTC films, we performed optical polarized microscopy (POM), Atomic Force Microscopy (AFM) and X-rays Diffraction (XRD) of the thin films.

1. Impact of preparation methods of CTC solutions on films

We tested three distinct methods for preparing the CTC solutions, with the parent compounds mixed in a 1:1 molar ratio and dissolved in a chlorobenzene:benzonitrile (5:1) solvent mixture at a final concentration of 18 mg mL^{-1}: (1) dissolving the parent compounds separately before mixing them (MET1), (2) mixing the parent compounds in a vial and dissolving them together (MET2), and (3) mixing the parent compounds, grinding them in a mortar, and subsequently dissolving the mixture (MET3). **Figure S14** and **Figure S15** report the morphological and XRD analysis respectively.

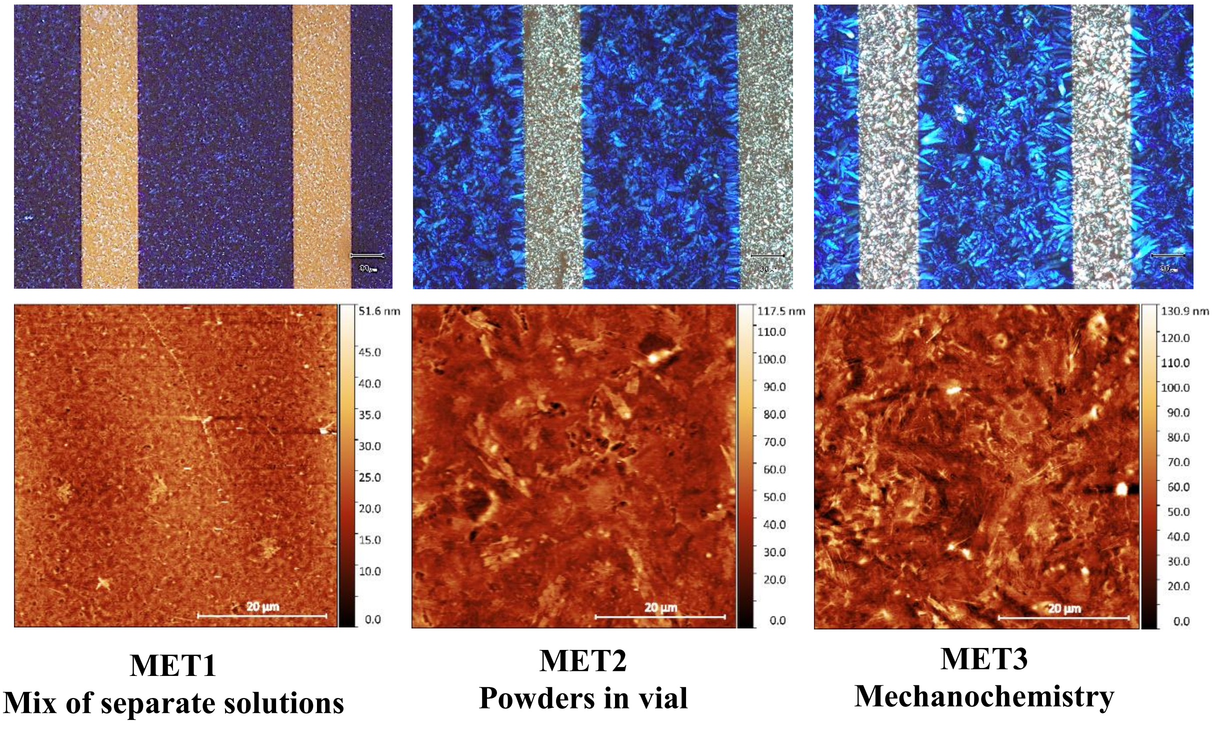

Figure S14. Top: POM images of CTC:PS (280 kDa) films deposited at 2 mm s^{-1} using three different methods (1, 2, and 3), showing the microstructure and morphology of the films. Bottom: Topographical images by AFM of the same CTC:PS (280 kDa) films, highlighting the differences in surface roughness and nanoscale features for each deposition method (1, 2, and 3).

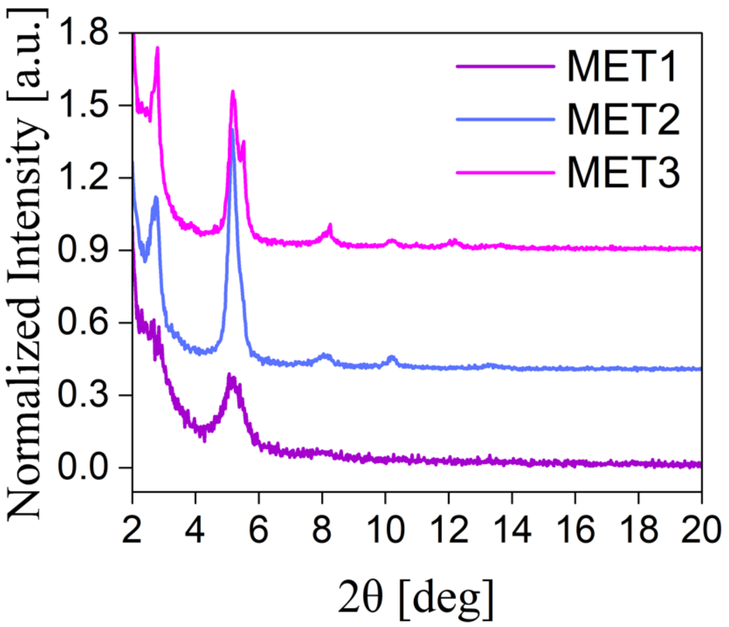

Figure S15. XRD analysis of CTC films blended with PS (280 kDa), deposited at 2 mm s^{-1} using three different solution preparation methods. The data reveal a substantial increase in crystallinity for films prepared using MET2 and MET3, while MET1 shows lower crystallinity.

2. Impact of deposition speed

Thin films of CTCs blended with PS (280 kDa) were deposited using both MET2 and MET3 at coating speeds of 0.8, 2, and 10 mm s^{-1}. The POM and AFM images, shown in **Figure S16**, demonstrate that for both methods, lower speeds result in greater film homogeneity, while higher speeds lead to a mixture of crystals with varying sizes. Among the two methods, MET2 consistently produces the most homogeneous films. XRD analysis reveals that the crystal structure remains unchanged across all conditions, as shown in **Figure S17**. Based on these results, we excluded the highest speed of 10 mm s^{-1} from further consideration. However, the data do not clearly indicate a preference between MET2 and MET3.

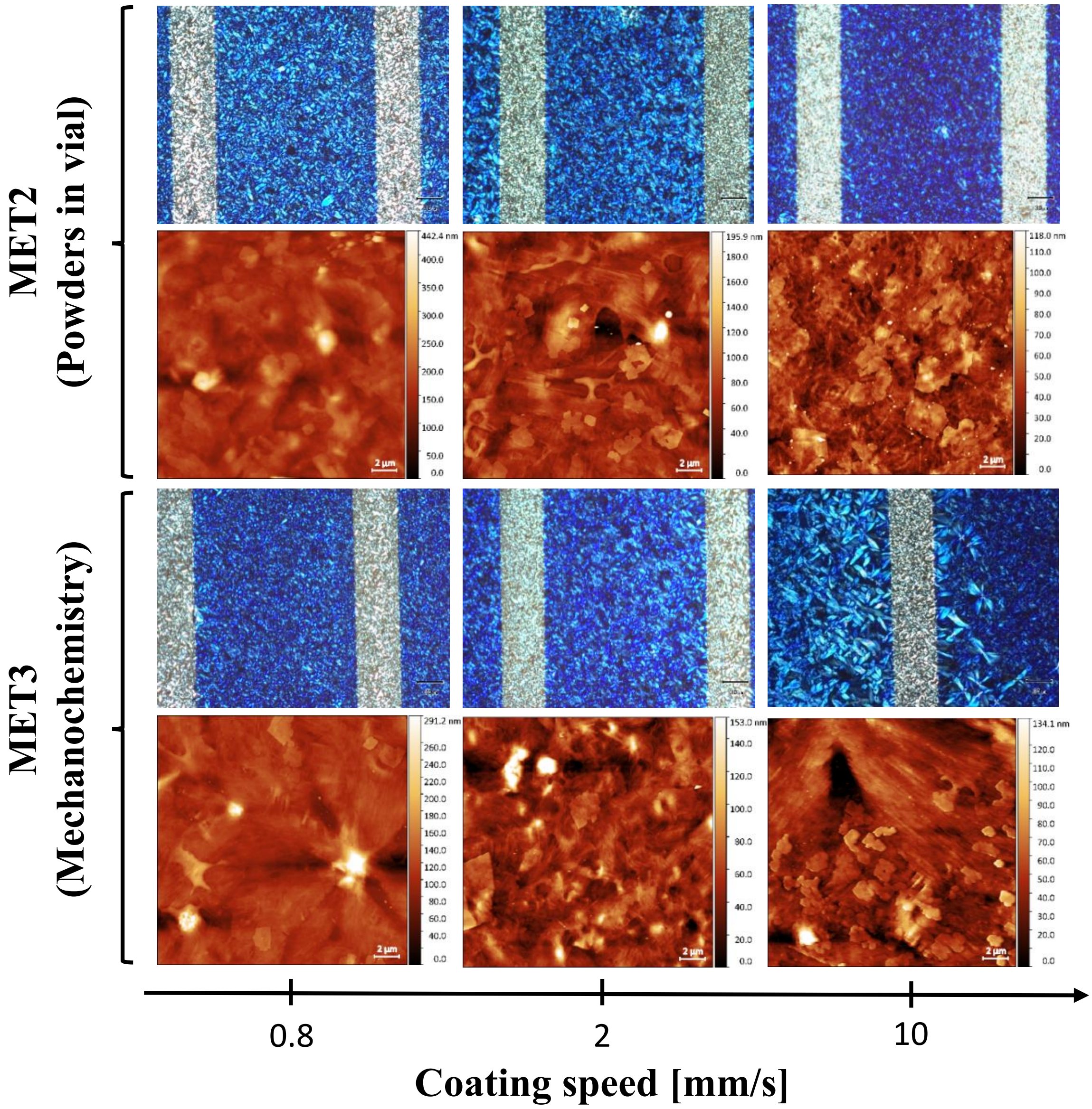

Figure S16. POM and AFM images of CTC:PS (280 kDa) films deposited at varying speeds (0.8, 2, and 10 mm s^{-1}) using MET2 and MET3. The first and second rows show POM and AFM images for MET2, while the third and fourth rows show POM and AFM images for MET3. As deposition speed increases, both homogeneity and uniformity degrade. At 0.8 mm s^{-1}, MET2 produces smaller, more uniform crystals, while MET3 generally shows a mixture of different crystal sizes and shapes, with larger spherulites intermixed with smaller crystals, accompanied by increased surface roughness.

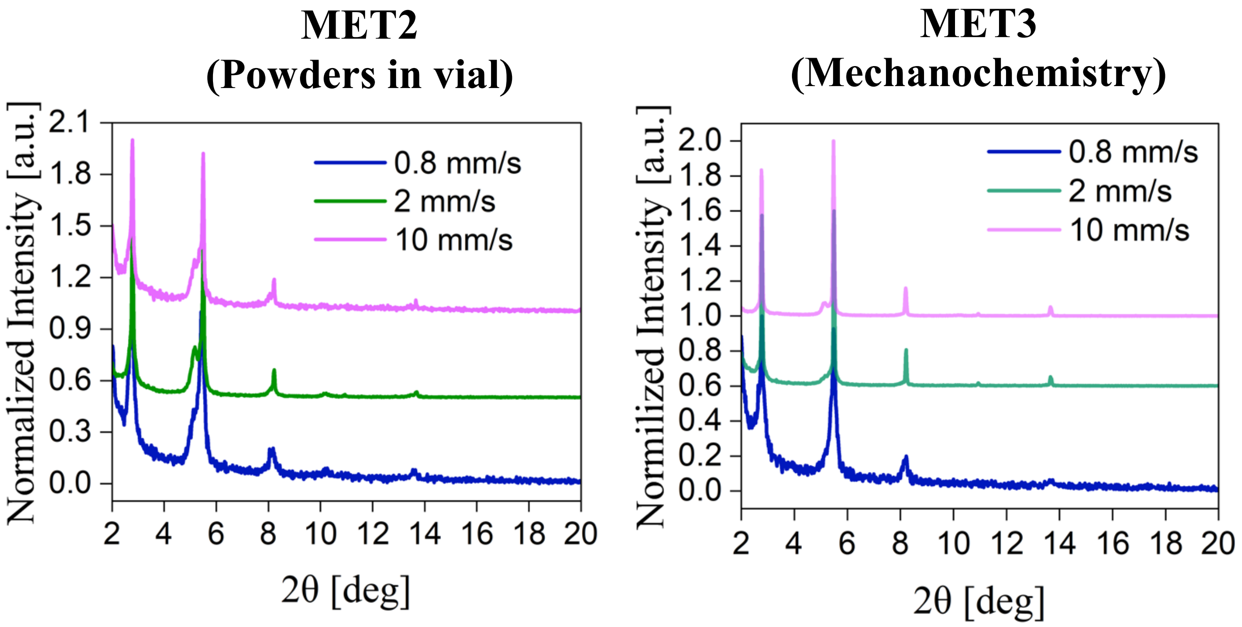

Figure S17. XRD analysis of CTC films blended with PS (280 kDa), deposited at varying speed (0.8, 2 and 10 mm s^{-1}) using MET2 and MET3 as solution preparation methods.

3. Impact of average-molecular weight of polystyrene

We tested different PS molecular weights (10 kDa, 100 kDa, and 280 kDa) using both MET2 and MET3. We report the films deposited at varying molecular weights for both 2 mm s^{-1} (**Figure S18**) and 0.8 mm s^{-1} (**Figure S19**) coating speeds. Films prepared at 2 mm s^{-1} with mechanochemistry (MET3) exhibited larger crystals and higher surface roughness. In contrast, films prepared using the powder-in-vial method (MET2) were more homogeneous. Additionally, lower coating speeds (0.8 mm/s) resulted in the best film uniformity for both methods. PS 10 kDa at 0.8 mm s^{-1} produced the smallest crystal structures and resulted in the most performing OFETs. XRD analysis (**Figure S20**) demonstrates that the crystal structures of the films were comparable across all conditions.

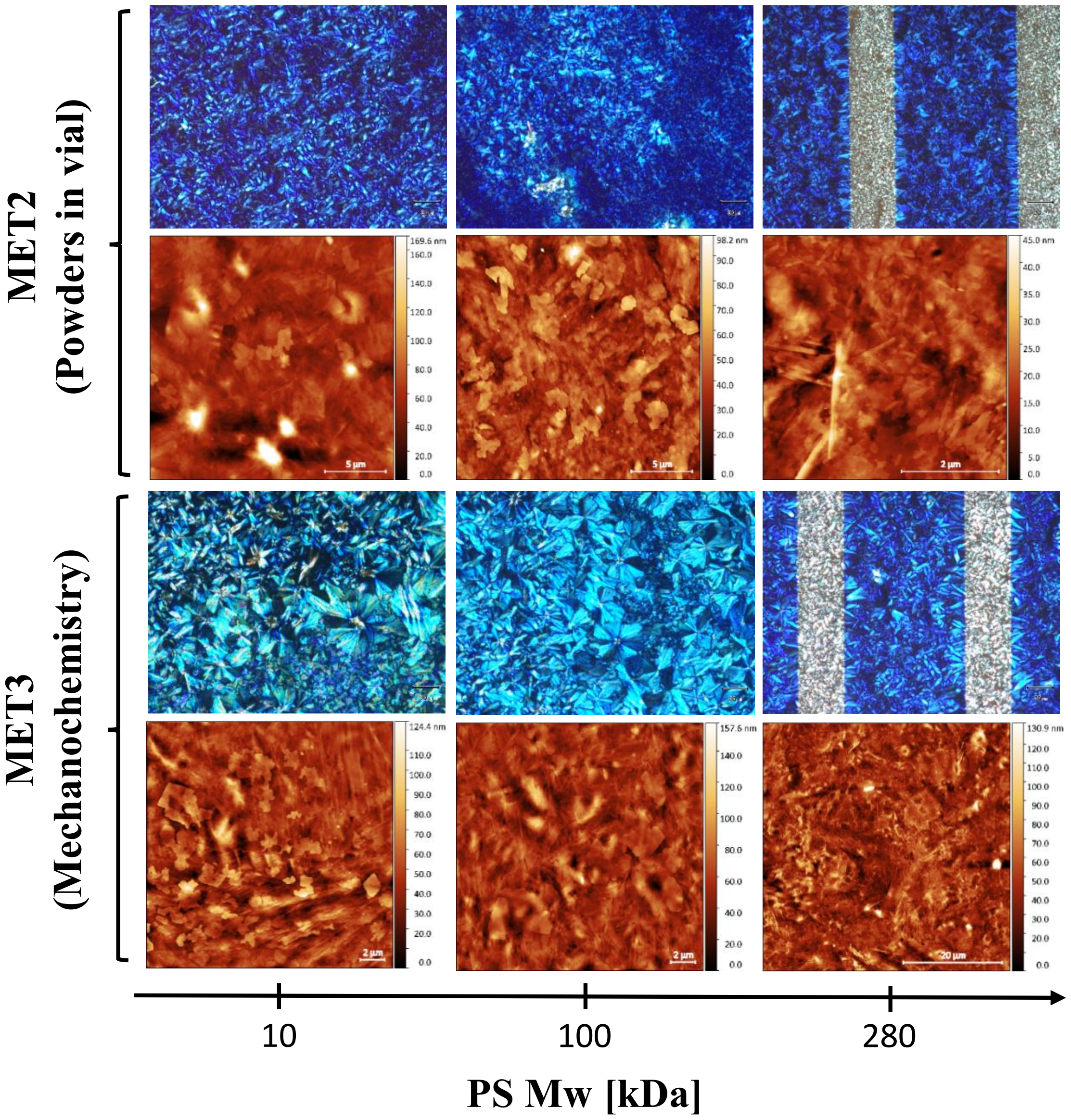

Figure S18. POM and AFM images of CTC:PS films with varying average molecular weights (10 kDa, 100 kDa, and 280 kDa), deposited at 2 mm s^{-1} using MET2 and MET3. The first and second rows show POM and AFM images for MET2, while the third and fourth rows show POM and AFM images for MET3. The films exhibit non uniform distribution of crystals except for PS 10 kDa MET2, with MET3 showing more pronounced crystal size variation compared to MET2.

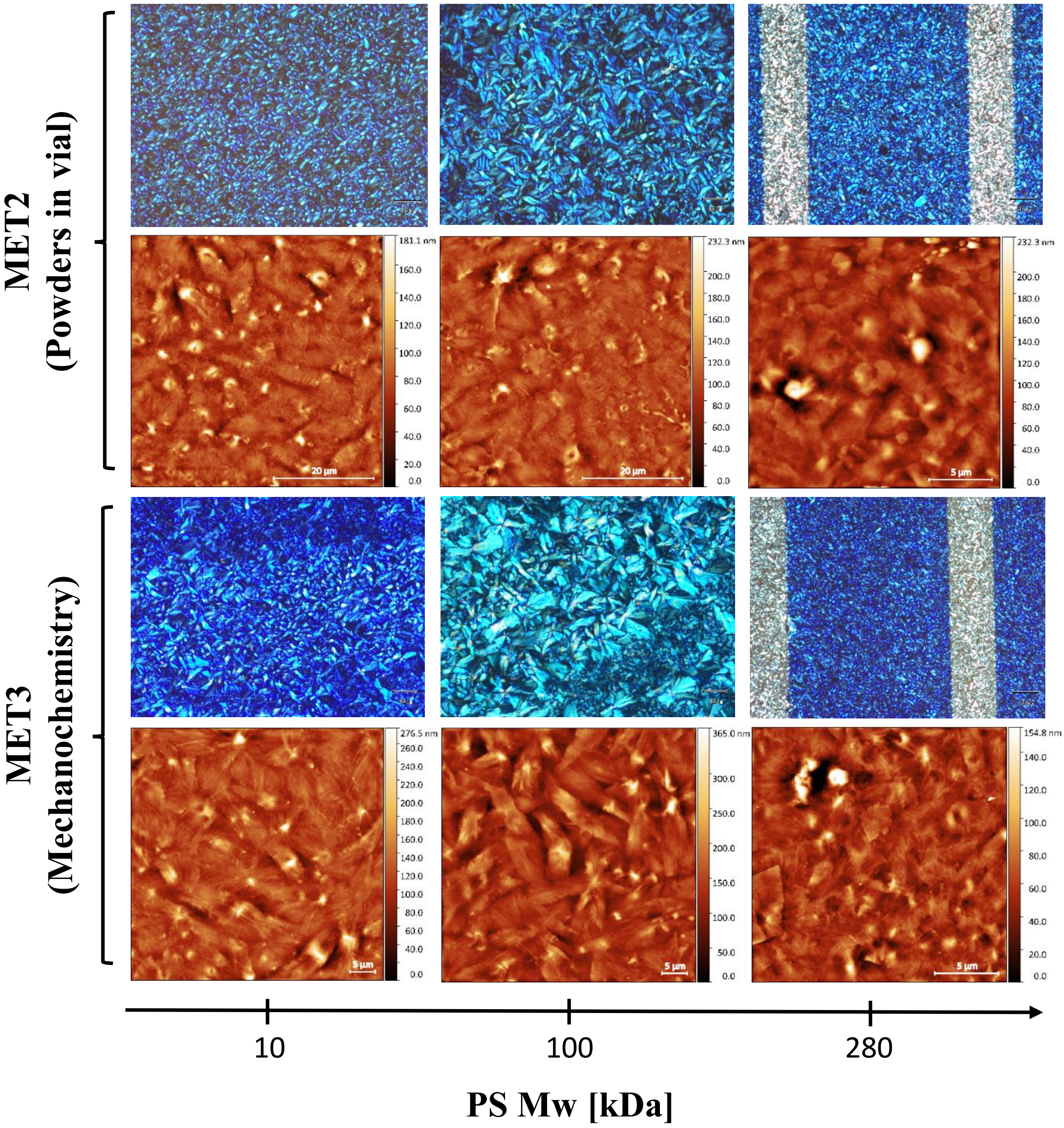

Figure S19. POM and AFM images of CTC:PS films with varying average molecular weights Mw (10 kDa, 100 kDa, and 280 kDa), deposited at 0.8 mm s^{-1} using MET2 and MET3. The first and second rows show POM and AFM images for MET2, while the third and fourth rows show POM and AFM images for MET3. At this lower deposition speed, the films display smaller, more uniform crystals, with a noticeable increase in crystal size and surface roughness for higher PS 100 kDa, particularly with MET3, which shows a more diverse mixture of crystal sizes.

a)

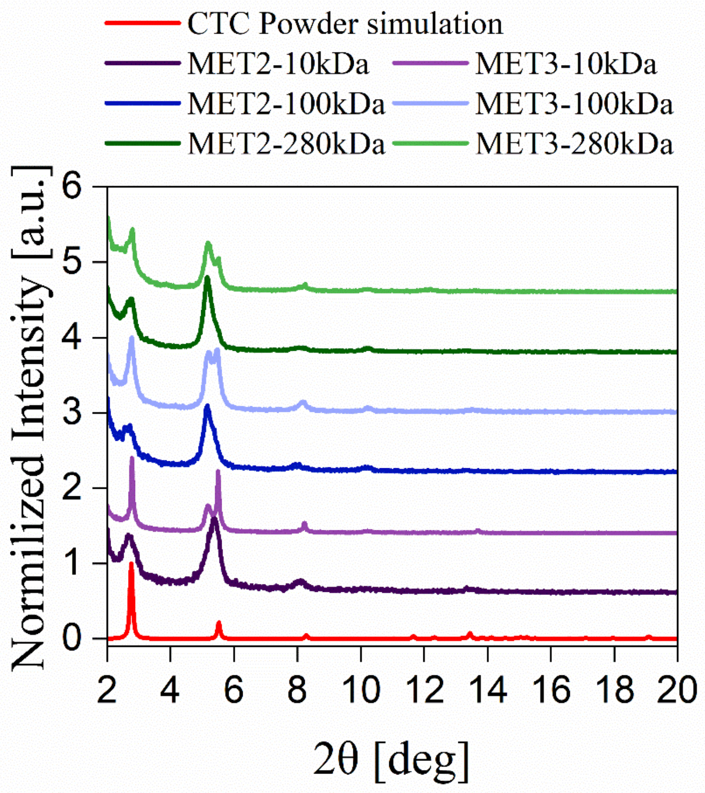

b)

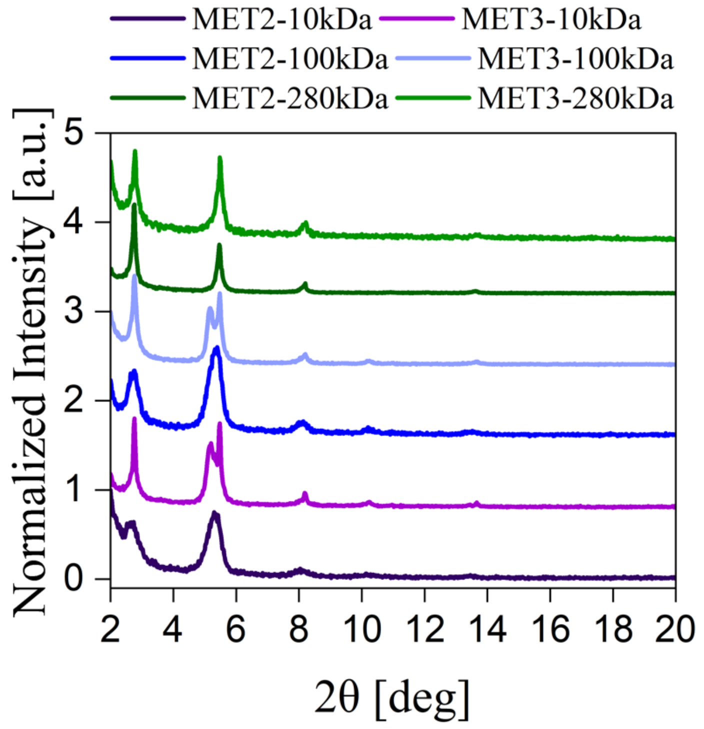

Figure S20. XRD analysis of CTC:PS films with varying PS average molecular weights (10 kDa, 100 kDa, and 280 kDa) deposited at (a) 2 mm s^{-1} and (b) 0.8 mm s^{-1} using MET2 and MET3.

References

(1) Yin, Y.; Xi, Z.; Yu, Q.; Gong, X.; Wang, H.; Yao, B.; Xu, H.; Shi, J.; Yin, L.; Yang, W.; Zhang, X.; Wei, Y.; Luo, X.; Fang, Z. Near-Infrared-II Balanced Ambipolar Phototransistors Realized by the Optimized Planar-Heterojunction Channel Layer and Charge-Transfer-Complex Photosensitive Layer. *Results Phys.* **2023**, *48*, 106456. https://doi.org/10.1016/j.rinp.2023.106456.

(2) Li, F.; Zheng, L.; Sun, Y.; Li, S.; Sun, L.; Yang, F.; Dong, W.; Zhang, X.; Hu, W. Cocrystal Engineering: Towards High-Performance near-Infrared Organic Phototransistors Based on Donor-Acceptor Charge Transfer Cocrystals. *Sci. China Chem.* **2023**, *66* (1), 266–272. https://doi.org/10.1007/s11426-022-1450-0.

(3) Luo, L.; Huang, W.; Ju, Z.; Mu, Z.; Wang, W.; Zhou, Y.; Zhang, J.; Huang, W. Charge-Transfer Pentacene/Benzothiadiazole Derivative Cocrystal for UV-to-NIR Large Range Responsive Phototransistors. *Org. Electron.* **2022**, *100*, 106363. https://doi.org/10.1016/j.orgel.2021.106363.

(4) Yang, B.; Wang, Y.; Li, L.; Zhang, J.; Wang, J.; Jiao, H.; Hao, D.; Guo, P.; Zeng, S.; Hua, Z.; Huang, J. High Performance Ternary Organic Phototransistors with Photoresponse up to 2600 Nm at Room Temperature. *Adv. Funct. Mater.* **2021**, *31* (40), 2103787. https://doi.org/10.1002/adfm.202103787.

(5) Iqbal, M. A.; Liaqat, A.; Hussain, S.; Wang, X.; Tahir, M.; Urooj, Z.; Xie, L. Ultralow-Transition-Energy Organic Complex on Graphene for High-Performance Shortwave Infrared Photodetection. *Adv. Mater.* **2020**, *32* (37), 2002628. https://doi.org/10.1002/adma.202002628.

(6) Iqbal, M. A.; Cui, M.; Liaqat, A.; faiz, R.; Hossain, M.; Wang, X.; Hussain, S.; Dang, C.; Liu, H.; Wen, W.; Wu, J.; Xie, L. Organic Charge Transfer Complexes on Graphene with Ultrahigh near Infrared Photogain. *Nanotechnology* **2019**, *30* (25), 254003. https://doi.org/10.1088/1361-6528/ab0608.

(7) Cui, M.; Guo, Y.; Zhu, Y.; Liu, H.; Wen, W.; Wu, J.; Cheng, L.; Zeng, Q.; Xie, L. Graphene–Organic Two-Dimensional Charge-Transfer Complexes: Intermolecular Electronic Transitions and Broadband Near-Infrared Photoresponse. *J. Phys. Chem. C* **2018**, *122* (13), 7551–7556. https://doi.org/10.1021/acs.jpcc.8b01408.

(8) Wang, C.; Ren, X.; Xu, C.; Fu, B.; Wang, R.; Zhang, X.; Li, R.; Li, H.; Dong, H.; Zhen, Y.; Lei, S.; Jiang, L.; Hu, W. N-Type 2D Organic Single Crystals for High-Performance Organic Field-Effect Transistors and Near-Infrared Phototransistors. *Adv. Mater.* **2018**, *30* (16), 1706260. https://doi.org/10.1002/adma.201706260.

(9) Liang, Y.; Lv, W.; Luo, X.; He, L.; Xu, K.; Zhao, F.; Huang, F.; Lu, F.; Peng, Y. A Comprehensive Investigation of Organic Active Layer Structures toward High Performance Near-Infrared Phototransistors. *Synth. Met.* **2018**, *240*, 44–51. https://doi.org/10.1016/j.synthmet.2018.03.016.

(10) Huang, F.; Li, Y.; Xia, H.; Zhang, J.; Xu, K.; Peng, Y.; Liu, G. Towards High Performance Broad Spectral Response Fullerene Based Photosensitive Organic Field-Effect Transistors with Tricomponent Bulk Heterojunctions. *Carbon* **2017**, *118*, 666–674. https://doi.org/10.1016/j.carbon.2017.03.091.

(11) Li, F.; Chen, Y.; Ma, C.; Buttner, U.; Leo, K.; Wu, T. High-Performance Near-Infrared Phototransistor Based on n-Type Small-Molecular Organic Semiconductor. *Adv. Electron. Mater.* **2017**, *3* (1), 1600430. https://doi.org/10.1002/aelm.201600430.

(12) Peng, Y.; Lv, W.; Yao, B.; Fan, G.; Chen, D.; Gao, P.; Zhou, M.; Wang, Y. High Performance near Infrared Photosensitive Organic Field-Effect Transistors Realized by

an Organic Hybrid Planar-Bulk Heterojunction. *Org. Electron.* **2013**, *14* (4), 1045–1051. https://doi.org/10.1016/j.orgel.2013.02.005.
(13) Ma, L.; Li, Z.; Chen, B.; Xue, P.; Wang, Z.; Wu, Y.; Zhan, X.; Liu, Y.; Chen, X. The Impact of Benzothiadiazole on the Optoelectronic Performance of Polymer/PC71BM Blend Films and Their Application in NIR Phototransistors. *Adv. Electron. Mater.* **2022**, *8* (7), 2101297. https://doi.org/10.1002/aelm.202101297.
(14) He, Z.; Han, J.; Du, X.; Cao, L.; Wang, J.; Zheng, C.; Lin, H.; Tao, S. Photomemory and Pulse Monitoring Featured Solution-Processed Near-Infrared Graphene/Organic Phototransistor with Detectivity of 2.4 × 1013 Jones. *Adv. Funct. Mater.* **2021**, *31* (37), 2103988. https://doi.org/10.1002/adfm.202103988.
(15) Li, Q.; Ran, Y.; Shi, W.; Qin, M.; Sun, Y.; Kuang, J.; Wang, H.; Chen, H.; Guo, Y.; Liu, Y. High-Performance near-Infrared Polymeric Phototransistors Realized by Combining Cross-Linked Polymeric Semiconductors and Bulk Heterojunction Bilayer Structures. *Appl. Mater. Today* **2021**, *22*, 100899. https://doi.org/10.1016/j.apmt.2020.100899.
(16) Jiang, X.; Lu, J.; Xue, D.; Wei, Y.; Zhang, Y.; Zhang, J.; Wang, Z.; Huang, L.; Chi, L. High Performance Near-Infrared Phototransistors via Enhanced Electron Trapping Effect. *Chem. Commun.* **2021**, *57* (91), 12123–12126. https://doi.org/10.1039/D1CC04828G.
(17) Lee, C.; Kim, H.; Kim, Y. Short-Wave Infrared Organic Phototransistors with Strong Infrared-Absorbing Polytriarylamine by Electron-Transfer Doping. *Npj Flex. Electron.* **2021**, *5* (1), 1–9. https://doi.org/10.1038/s41528-021-00105-z.
(18) Wang, G.; Huang, K.; Liu, Z.; Du, Y.; Wang, X.; Lu, H.; Zhang, G.; Qiu, L. Flexible, Low-Voltage, and n-Type Infrared Organic Phototransistors with Enhanced Photosensitivity via Interface Trapping Effect. *ACS Appl. Mater. Interfaces* **2018**, *10* (42), 36177–36186. https://doi.org/10.1021/acsami.8b12009.
(19) Rim, Y. S.; Ok, K.-C.; Yang, Y. M.; Chen, H.; Bae, S.-H.; Wang, C.; Huang, Y.; Park, J.-S.; Yang, Y. Boosting Responsivity of Organic–Metal Oxynitride Hybrid Heterointerface Phototransistor. *ACS Appl. Mater. Interfaces* **2016**, *8* (23), 14665–14670. https://doi.org/10.1021/acsami.6b02814.
(20) Han, H.; Nam, S.; Seo, J.; Lee, C.; Kim, H.; Bradley, D. D. C.; Ha, C.-S.; Kim, Y. Broadband All-Polymer Phototransistors with Nanostructured Bulk Heterojunction Layers of NIR-Sensing n-Type and Visible Light-Sensing p-Type Polymers. *Sci. Rep.* **2015**, *5* (1), 16457. https://doi.org/10.1038/srep16457.
(21) Xu, H.; Li, J.; K. Leung, B. H.; Y. Poon, C. C.; S. Ong, B.; Zhang, Y.; Zhao, N. A High-Sensitivity near-Infrared Phototransistor Based on an Organic Bulk Heterojunction. *Nanoscale* **2013**, *5* (23), 11850–11855. https://doi.org/10.1039/C3NR03989G.
(22) Zhong, W.; Wang, X.; Wang, W.; Song, Z.; Tang, Y.; Chen, B.; Yang, T.; Liang, Y. Fast Near-Infrared Organic Photodetectors with Enhanced Detectivity by Molecular Engineering of Acceptor Materials. *Adv. Sci.* **2025**, *12* (3), 2410332. https://doi.org/10.1002/advs.202410332.
(23) Yang, M.; Yin, B.; Hu, G.; Cao, Y.; Lu, S.; Chen, Y.; He, Y.; Yang, X.; Huang, B.; Li, J.; Wu, B.; Pang, S.; Shen, L.; Liang, Y.; Wu, H.; Lan, L.; Yu, G.; Huang, F.; Cao, Y.; Duan, C. Sensitive Short-Wavelength Infrared Photodetection with a Quinoidal Ultralow Band-Gap n-Type Organic Semiconductor. *Chem* **2024**, *10* (5), 1425–1444. https://doi.org/10.1016/j.chempr.2024.01.002.
(24) Zhang, H.; Mao, R.; Yuan, L.; Wang, Y.; Liu, W.; Wang, J.; Tai, H.; Jiang, Y. Near-Infrared Organic Photodetectors with Spectral Response over 1200 Nm Adopting a Thieno[3,4-c]Thiadiazole-Based Acceptor. *ACS Appl. Mater. Interfaces* **2024**, *16* (7), 9088–9097. https://doi.org/10.1021/acsami.3c15902.
(25) Li, T.; Hu, G.; Tao, L.; Jiang, J.; Xin, J.; Li, Y.; Ma, W.; Shen, L.; Fang, Y.; Lin, Y. Sensitive Photodetection below Silicon Bandgap Using Quinoid-Capped Organic

Semiconductors. *Sci. Adv.* **2023**, *9* (13), eadf6152. https://doi.org/10.1126/sciadv.adf6152.
(26) Jacoutot, P.; Scaccabarozzi, A. D.; Nodari, D.; Panidi, J.; Qiao, Z.; Schiza, A.; Nega, A. D.; Dimitrakopoulou-Strauss, A.; Gregoriou, V. G.; Heeney, M.; Chochos, C. L.; Bakulin, A. A.; Gasparini, N. Enhanced Sub-1 eV Detection in Organic Photodetectors through Tuning Polymer Energetics and Microstructure. *Sci. Adv.* **2023**, *9* (23), eadh2694. https://doi.org/10.1126/sciadv.adh2694.
(27) Park, I.; Kim, C.; Kim, R.; Li, N.; Lee, J.; Kwon, O. K.; Choi, B.; Ng, T. N.; Leem, D.-S. High Performance Shortwave Infrared Organic Photodetectors Adopting Thiadiazole Quinoxaline-Based Copolymers. *Adv. Opt. Mater.* **2022**, *10* (19), 2200747. https://doi.org/10.1002/adom.202200747.
(28) Xu, C.; Liu, P.; Feng, C.; He, Z.; Cao, Y. Organic Photodetectors with High Detectivity for Broadband Detection Covering UV-Vis-NIR. *J. Mater. Chem. C* **2022**, *10* (15), 5787–5796. https://doi.org/10.1039/D2TC00525E.
(29) Zhong, Z.; Peng, F.; Ying, L.; Yu, G.; Huang, F.; Cao, Y. Ternary Organic Photodiodes with Spectral Response from 300 to 1200 Nm for Spectrometer Application. *Sci. China Mater.* **2021**, *64* (10), 2430–2438. https://doi.org/10.1007/s40843-020-1639-x.
(30) Strobel, N.; Droseros, N.; Köntges, W.; Seiberlich, M.; Pietsch, M.; Schlisske, S.; Lindheimer, F.; Schröder, R. R.; Lemmer, U.; Pfannmöller, M.; Banerji, N.; Hernandez-Sosa, G. Color-Selective Printed Organic Photodiodes for Filterless Multichannel Visible Light Communication. *Adv. Mater.* **2020**, *32* (12), 1908258. https://doi.org/10.1002/adma.201908258.
(31) Huang, J.; Lee, J.; Vollbrecht, J.; Brus, V. V.; Dixon, A. L.; Cao, D. X.; Zhu, Z.; Du, Z.; Wang, H.; Cho, K.; Bazan, G. C.; Nguyen, T.-Q. A High-Performance Solution-Processed Organic Photodetector for Near-Infrared Sensing. *Adv. Mater.* **2020**, *32* (1), 1906027. https://doi.org/10.1002/adma.201906027.
(32) Hu, X.; Dong, Y.; Huang, F.; Gong, X.; Cao, Y. Solution-Processed High-Detectivity Near-Infrared Polymer Photodetectors Fabricated by a Novel Low-Bandgap Semiconducting Polymer. *J. Phys. Chem. C* **2013**, *117* (13), 6537–6543. https://doi.org/10.1021/jp4001237.
(33) Ng, T.-W.; Yang, Q.-D.; Mo, H.-W.; Lo, M.-F.; Zhang, W.-J.; Lee, C.-S. Wide-Spectral Photoresponse of Black Molybdenum Oxide Photodetector via Sub-Bandgap Electronic Transition. *Adv. Opt. Mater.* **2013**, *1* (10), 699–702. https://doi.org/10.1002/adom.201300220.
